# *In vitro* and *in vivo* investigation of a zonal microstructured scaffold for osteochondral defect repair

**DOI:** 10.1016/j.biomaterials.2022.121548

**Published:** 2022-05-02

**Authors:** Joseph A.M. Steele, Axel C. Moore, Jean-Philippe St-Pierre, Seth D. McCullen, Adam J. Gormley, Conor C. Horgan, Cameron RM. Black, Christoph Meinert, Travis Klein, Siamak Saifzadeh, Roland Steck, Jiongyu Ren, Maria A. Woodruff, Molly M. Stevens

**Affiliations:** a Department of Materials, Imperial College London, SW7 2AZ, UK; b Department of Bioengineering, Imperial College London, SW7 2AZ, UK; c Institute of Biomedical Engineering, Imperial College London, SW7 2AZ, UK; d Division of Biomaterials and Regenerative Medicine, Department of Medical Biochemistry and Biophysics, Karolinska Institute, Stockholm, SE 171 77, Sweden; e Bone and Joint Research Group, Centre for Human Development, Stem Cells and Regeneration, Developmental Origins of Health and Disease, Institute of Developmental Sciences, University of Southampton Medical School, Southampton, SO16 6YD, UK; f Institute of Health and Biomedical Innovation, Queensland University of Technology, Brisbane, Australia; g Medical Engineering Research Facility, Queensland University of Technology, Brisbane, Australia; h ARC Training Centre in Additive Biomanufacturing, Brisbane, Australia

**Keywords:** Microstructured scaffold, Polycaprolactone, Zonal articular cartilage, Cartilage tissue engineering, Osteochondral defect repair, MaioRegen™

## Abstract

Articular cartilage is comprised of zones that vary in architecture, extracellular matrix composition, and mechanical properties. Here, we designed and engineered a porous zonal microstructured scaffold from a single biocompatible polymer (poly [ϵ-caprolactone]) using multiple fabrication strategies: electrospinning, spherical porogen leaching, directional freezing, and melt electrowriting. With this approach we mimicked the zonal structure of articular cartilage and produced a stiffness gradient through the scaffold which aligns with the mechanics of the native tissue. Chondrocyte-seeded scaffolds accumulated extracellular matrix including glycosaminoglycans and collagen II over four weeks *in vitro.* This prompted us to further study the repair efficacy in a skeletally mature porcine model. Two osteochondral lesions were produced in the trochlear groove of 12 animals and repaired using four treatment conditions: (1) microstructured scaffold, (2) chondrocyte seeded microstructured scaffold, (3) MaioRegen™, and (4) empty defect. After 6 months the defect sites were harvested and analyzed using histology, micro computed tomography, and Raman microspectroscopy mapping. Overall, the scaffolds were retained in the defect space, repair quality was repeatable, and there was clear evidence of osteointegration. The repair quality of the microstructured scaffolds was not superior to the control based on histological scoring; however, the lower score was biased by the lack of histological staining due to the limited degradation of the implant at 6 months. Longer follow up studies (e.g., 1 yr) will be required to fully evaluate the efficacy of the microstructured scaffold. In conclusion, we found consistent scaffold retention, osteointegration, and prolonged degradation of the microstructured scaffold, which we propose may have beneficial effects for the long-term repair of osteochondral defects.

## 1 Introduction

Healthy articular cartilage supports load transmission and near-frictionless joint articulation between contacting surfaces [1–4]. The harsh demands placed on articular joints (cutting movements, jumping, descending stairs, etc.) coupled with the poor intrinsic repair of cartilage can lead to tissue damage and eventually arthritis of the joint.

Significant effort has been invested into developing repair scaffolds for cartilage defects (e.g., hydrogels [5–8], mats [9,10], foams [11], and open latticework [12]). One of the main areas of development has been mimicking the structural, morphological, chemical, and cellular gradients found in native articular cartilage [13–15]. For example, the generally high-water content of hydrogels has made them a popular choice for mimicking proteoglycans, while 3D printed lattices and electrospun meshes have been used to mimic the fibrous collagen architecture. Reviews of zonally organized and functionally graded scaffolds for articular cartilage regeneration highlight the wide array of approaches that have been investigated [16–19]. Previous work by the authors produced multi-layered electrospun and fiber-foam composite scaffolds that supported tissue formation and provided mechanical anisotropy *in vitro* [20–22]. Related systems include layered collagen-hyaluronic acid foams [23], poly (L-lactide--co-ε-caprolactone)-collagen fiber-foam-mineral scaffolds [24], directionally frozen poly (D,L-lactide) cellulose foams [25], and commercial products such as Novocart® and Chondro-Gide®.

While many solutions for isolated cartilage lesions are under development, there remain few examples for osteochondral defects, which involve deterioration of both the cartilage and the subchondral bone. Notable exceptions to this are TruFit CB® and MaioRegen™, which use a bone scaffold in addition to the chondrogenic zone. Both commercially available scaffolds showed promise in early clinical trials; however, TruFit CB® is no longer available due to highly variable longitudinal outcomes [26].

**Fig. 1:**
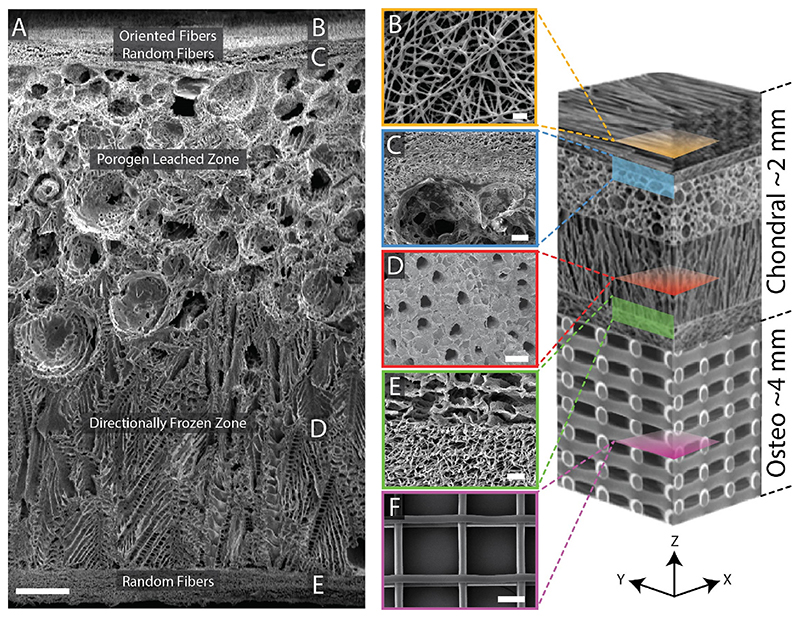
Scanning electron micrographs of a zonal microstructured scaffold. (A) Cross-section of the complete scaffold showing each unique zone. (B) Partially fused PCL fibers used to adhere the electrospun mat to the underlying foam using residual solvent (top-down image). (C) A cross-sectional view of the porogen-electrospun interface. (D) Vertical channels through the directionally frozen foam (top-down image). (E) A cross-sectional view of the directionally frozen-electrospun interface. (F) Melt-electrowritten osteo component (top-down image). The osteo component consisted of 20 μm diameter fibers stacked at 200 μm intervals in a 90-degree laydown pattern. Figure at right is a conceptual schematic of the zonal microstructured osteochondral scaffold, features are not proportionally represented. Scale bars for images A, B, C, D, E, and F are 250, 10, 50, 100, 25, and 100 μm, respectively.

In this study, we developed a zonal microstructured scaffold for osteochondral defect repair. The design was bioinspired to mimic the multizonal microstructure of articular cartilage and built upon the outcomes of our previous work; specifically, the beneficial effect of multiple fiber populations for *in vitro* cartilage formation [9], the role of fiber alignment in surface damage mitigation [27], and the role of pore size in fiber-porogen scaffolds [28]. The scaffold was composed of aligned electrospun fibers in the superficial zone, a porogen leached intermediate zone, a directionally frozen deep zone, and a fibrous osteochondral interface (Fig. 1). The scaffold demonstrated mechanical anisotropy and supported chondrocytes and matrix deposition in an *in vitro* model. Following this, we assessed the microstructured scaffold in a porcine osteochondral defect model and included a thermally fused polymer bone mimic. Poly (ϵ-caprolactone) (PCL) was selected as the optimal scaffold material for our application as it can be processed with multiple fabrication techniques, has a compressive modulus of ~0.4 GPa, is used in FDA approved devices (e.g., Osteomesh™), and is biocompatible and biodegradable [29]. The results demonstrate that the microstructured scaffolds remained visually intact after 6 months implantation and exhibited matrix deposition, integration with the subchondral bone, and a flush articular surface.

## 2 Materials and methods

All chemicals were acquired from Sigma Aldrich, UK and all cell-culture materials were acquired from Invitrogen, UK and used as received, unless otherwise specified.

### 2.1 Scaffold fabrication

The composite scaffolds (2 mm thick) were produced in a step-wise manner, first generating a porogen-leached/directionally-frozen integrated foam then electrospinning fibrous layers onto the top and bottom faces of the scaffold. The detailed protocol is included in the Supplemental Methods and Figure S1.

Prior to scaffold fabrication, thermoset gelatin microspheres were produced. An emulsion of 20% (w/v) porcine skin gelatin type A in sunflower oil stabilized by 0.5% (v/v) Tween 20 was thermoset at 500 rpm in an ice bath and dehydrated with cold acetone washes. Microspheres were sieved to collect 100–300 μm diameter particles and stored under dry conditions. Gelatin microspheres were resuspended at 71% (w/v) in 8% (w/v) 80 kDa PCL in dioxane, the resulting slurry was deposited as a 1 mm-thick layer on a copper plate within an 80 × 80 × 2 mm PDMS mold and allowed to dry. An additional 1 mm of PCL-dioxane was then deposited onto the dried PCL-gelatin microspheres mixture and directionally frozen on a − 20 °C PolarBear Plus cold-plate (Cambridge Reactor Design, UK). The phase separated dioxane crystal regions were removed by lyophilization, and the gelatin was dissolved in water at 37 °C followed by PBS.

Electrospun PCL fibers were deposited onto both sides of the foam from a solution of 12% (w/v) PCL in 1,1,1,3,3,3-hexafluoroisopropanol (HFIP) at 2 mL/h, 11 kV, via a 19-gauge blunt needle. The foams were mounted to an electrically grounded mandrel 100 mm wide and 200 mm in diameter. The mandrel was rotated at 100 rpm (1 m/s surface speed) to collect random fibers, and 2000 rpm (21 m/s surface speed) to collect oriented fibers. The first hour of spinning was performed at a tip-to-target distance of 50 mm and 100 rpm to promote adhesion between the layers. The second hour of spinning was performed at 100 mm and 100 rpm to create a region of random fibers. The final 6 h of spinning was performed at 100 mm with rotational speeds of 100 rpm for the bottom (osteochondral interface) and 2000 rpm for the top of the scaffold (articular surface). The electrospinning produced a ~100 μm thick layer on the top and bottom of the scaffold giving a final thickness of 2 mm. Note that the scaffold in Fig. 1A is representative of the fabrication process but was designed for thicker cartilage applications (2.6 mm thick).

The porosity of the final scaffolds was measured by relative density. Dry scaffolds were cut into cylindrical sections and measured in triplicate for diameter and height using digital calipers (±0.01 mm resolution). The scaffolds were then massed on an analytical balance (±0.01 mg resolution) and the apparent density computed. The ratio of the apparent density to the known density for 80 kDa PCL (1.14 g/cm^3^) gave the relative density. Subtracting the relative density from 1 gave the porosity.

### 2.2 Scanning electron microscopy

Scaffolds were prepared by freeze-fracture in liquid nitrogen and coated with ~100 Å of gold in an Emitech K550 sputter coater. Images were obtained using a JEOL 5610 scanning electron microscope at an accelerating voltage of 10–15 kV.

Pore size was measured from electron micrographs using ImageJ and the built-in Analyze Particles function. Empirically determined lower bounds were established for each zone to eliminate noise.

### 2.3 Mechanical characterization

Compression testing of complete scaffolds and the individual zones was performed using a TA Electroforce® 3200 Series II test instrument equipped with a 5 lbf (22 N) tension-compression load cell under ambient conditions. Samples 6 mm in diameter and 2 mm thick were measured by digital calipers and tested hydrated in unconfined compression. For uniaxial compression testing, samples were pre-loaded to 0.05 N and compressed to 10% strain at a crosshead speed of 0.5% strain/min. A linear regression was fit from 0 to 5% and 5–10% strain to quantify the compressive modulus.

Dynamic mechanical analysis was performed in unconfined compression at 0.1, 1, and 10 Hz under dry and hydrated conditions. Samples were first compressed to a mean strain target (5, 10, 15, and 20%) before the addition of a 5% dynamic strain. The complex modulus (E*) was quantified for each frequency (0.1, 1, and 10 Hz) and mean strain (5, 10, 15, and 20%) for 3 samples. The complex modulus was calculated as $E^{*}=\left(F_{amp}\cdot t_{0}\right)/\left(\delta_{amp}\cdot \pi \cdot R_{0}^{2}\right)$, where *F_amp_* and *δ_amp_* are the force and deformation amplitudes, and *t_0_* and *R_0_* are the thickness and radius of the unstressed cylindrical sample. To determine the strain evolution over time, one sample was exposed to a 1 Hz cyclic compression test for 12 h under a 0.8 N force (28 kPa stress) amplitude.

Strain partitioning was quantified by compressing the bonded porogen leached and directionally frozen foam over a fluorescent microscope. The foam was first hydrated in a fluorescent PBS solution containing 4.55 × 10^7^ fluorescent beads/ml (Polysciences Inc.). The specimen was then compressed from 0 to 25% strain in 5% strain increments. Images were captured and post processed to quantify zonal deformation and strain.

### 2.4 In vitro culture of bovine chondrocytes in scaffolds

Primary bovine chondrocytes were isolated from cartilage excised from the stifle joints of approximately 1-year old calves with sequential incubation in pronase (0.2% w/v) and collagenase (0.04% w/v) and expanded to passage 2, as previously described [20]. Scaffolds (6 mm diameter) were sterilized by a 15 min incubation in 1000 ppm peracetic acid in 20% (v/v) ethanol, washed in sterile water, dried, and stored under sterile conditions before seeding. Ten million chondrocytes were resuspended in 50 μL of expansion medium. A single 45 μL bolus was injected into the porogen leached foam. The small pore size of the fibrous zone inhibits cellular migration; therefore, the superficial zone was seeded with the remaining 5 μL drop of cell concentrate. Scaffolds were incubated for 2 h at 37 °C and 100% RH before immersing in serum-free chondrogenic medium composed of Dulbecco’s modified Eagle medium (DMEM) (4.5 g/L glucose), 1% (v/v) insulin-transferrin-selenium (ITS) (100x), 1% (v/v) penicillin-streptomycin (P/S) (100x), 1% (v/v) Non-Essential Amino Acids (NEAA) (100x), 1% (v/v) HEPES (100x), 1% (v/v) GlutaMAX (100x), 0.4 mM L-Proline, 0.1 M dexamethasone, 0.1 mM ascorbic acid, and 10 ng/mL Recombinant Human TGF-β3 (R&D Systems, USA). Medium was changed every 48 h. ITS, dexamethasone, ascorbic acid, and TGF-β3 were added fresh during media changes.

### 2.5 Biochemical assessment

At weeks 0, 1, 2, and 4, scaffolds were digested for 24 h with 2.5 units papain/mL, 5 mM cysteine HCl, and 5 mM EDTA in sterile PBS at 60 °C. DNA content was quantified by the Quant-iT™ PicoGreen™ assay using a calibration curve of calf-thymus DNA. Sulphated glycosaminoglycan (sGAG) content was quantified using a dimethylmethylene blue (DMMB) assay at pH 1.5 [30,31]. Briefly, a DMMB solution was prepared by dissolving 16 mg DMMB in 5 mL ethanol and diluting to 1 L in distilled water with 40 mM NaCl and glycine. The solution was adjusted to pH 1.5 with 3 M HCl and filtered to remove precipitates. Samples from papain digest were diluted to 1:10 with papain digest buffer. 20 μL of diluted sample and 200 μL of DMMB solution were added to a 96-well plate and read immediately at 525 nm. sGAG was quantified using a 2–250 μg/mL standard curve of chondroitin-6-sulphate in papain digest buffer, run in duplicate in every plate. Samples were run in technical duplicate, with three replicates for each condition and time point. In the instances where the scaffolds were separated by zone, the electrospun layers were peeled from either side of the foam with forceps and a sterile blade was used to separate the porogen and directionally frozen foams. Each zone was then processed following the above protocol.

### 2.6 Gene expression

RNA was extracted from samples using a RNeasy kit (QIAGEN, UK) and 200 ng RNA was transcribed to cDNA via the QuantiTect Reverse Transcription kit (QIAGEN, UK). Gene expression was analyzed relative to the reference gene 18 S using the QuantiTect SYBR Green real time quantitative PCR kit on a StepOnePlus™ Real-Time PCR System (Applied Biosystems, UK). The primers used and their efficiencies are listed in Table S1. Genes are expressed as a fold increase over week 0 using the 2^−ΔΔCq^ method [32]. Multiple approaches were investigated to disrupt, dissolve, and macerate the scaffold samples in lysis buffer (RLT, QIAGEN) or phenol-chloroform (TRIzol®, Life Technologies) and extract the RNA with QIAGEN Cell Shredder and RNeasy Purification kits. Ultimately, it was determined that mincing the scaffolds with a scalpel and vortexing in RLT supplemented with β-mercaptoethanol with RNeasy purification, while omitting the cell-shredder preparatory columns, was the most reliable method for extraction of RNA.

### 2.7 In vitro histological assessment

Samples were fixed in 4% formaldehyde and dehydrated in an ethanol series for embedding in polyester wax at 45 °C. Low temperature embedding was used to retain the PCL within the histological sections [20]. Blocks were cut at 10 μm, dewaxed in Histo-Clear™, and stained with hematoxylin and eosin, Alcian blue, and picrosirius red to visualize cells and tissue, proteoglycans, and collagen, respectively.

### 2.8 Osteochondral scaffold fabrication

For *in vivo* implantation, an osteochondral scaffold was formed by thermally fusing the microstructured scaffold to a PCL bone scaffold. The bone scaffold was produced by the technique of melt-electrowriting [33]. The 4 mm thick scaffold was formed from 20 μm diameter fibers spaced 200 μm apart in a 90° pattern. The PCL (Perstorp CAPA 6400, 37 kDa) was heated to 73 °C and extruded at 40 μL/h from an 18G needle charged to +3.2 kV. A computer-controlled collector plate, charged to −3.2 kV, translated the platform across all 3 axes (X, Y, Z). The two components were thermally fused, producing a 6 mm diameter by 6 mm tall osteochondral scaffold. Scaffolds were sterilized by a 15 min incubation in 1000 ppm peracetic acid in 20% (v/v) ethanol, washed in sterile water, dried, and stored under sterile conditions before implantation.

### 2.9 Porcine chondrocyte isolation and expansion

Osteochondral plugs were obtained from porcine defects produced for acellular experimental groups and controls. Tissue plugs were stored for up to 24 h in expansion media containing DMEM with 1.0 g/L glucose, 1% (v/v) P/S, 1% (v/v) NEAA, 1% (v/v) HEPES, 0.4 mM L-Proline, 0.1 mM ascorbic acid, and 10% (v/v) fetal bovine serum. Cartilage was excised from the subchondral bone, pooled, and digested at 37 °C in 0.15% (w/v) collagenase type II (Worthington, Australia) in DMEM overnight in a sterile spinner flask at 60 rpm. The digest was filtered using a 100 μm cell strainer to remove extracellular matrix (ECM) debris, washed in expansion media, and plated in tissue culture flasks. Chondrocytes were expanded to passage 2 and used directly. This degree of expansion matches the *in vitro* validation and is within the range used clinically for autologous chondrocyte implantation and matrix-induced autologous chondrocyte implantation procedures (e.g., Cartipatch®, Hyalograft®C, and MACI®) [34].

### 2.10 Scaffold seeding and culture

The microstructured osteochondral scaffolds were used directly as acellular implants or pre-seeded with the allogenic chondrocyte population. Seeding was performed in a similar manner to that of the *in vitro* scaffolds. Briefly, passage 2 chondrocytes were suspended with trypsin and rinsed in serum-free chondrogenic media composed of DMEM (4.5 g/L glucose), 1% (v/v) ITS, 1% (v/v) P/S, 1% (v/v) NEAA, 1% (v/v) HEPES, 1% (v/v) GlutaMAX, 0.4 mM L-Proline, 0.1 M dexamethasone, 0.1 mM ascorbic acid, and 10 ng/mL TGF-β3. Viability was quantified by a hemocytometer and concentrated to 2 × 10^5^ cells/μL. Sterile scaffolds were rinsed three times in sterile PBS prior to cell seeding. Cells were injected into the scaffolds in a 45 μL bolus (9 × 10^6^ cells) via a 25G needle into the isotropic foam zone. A further 5 μL drop (1 × 10^6^ cells) of the cell suspension was deposited onto the top of the scaffold. The scaffold was incubated at 37 °C, 5% CO_2_, and 100% RH for 2 h. The scaffolds were then suspended in chondrogenic media and cultured for 1 week with media changes every 2 days.

On the day of implantation, scaffolds were rinsed and transported in sterile PBS, with no more than 15 min between removal from the incubator and implantation.

### 2.11 MaioRegen™ preparation

MaioRegen™ Prime (JRI Orthopaedics, UK) was purchased in the form of a sterile 35 mm × 35 mm x 6 mm sheet. Control scaffolds were cut to 6 mm diameter and stored with the acellular osteochondral scaffolds under sterile conditions until implantation.

### 2.12 Surgical implantation

All procedures were approved by the Queensland University of Technology Animal Ethics Committee (Animal Research Ethics Approval Certificate: 1,400,000,378). All work was performed at the Queensland University of Technology Medical Engineering Research Facility under GLP protocols. Twelve female pigs (60–97 kg, mean 78 kg) were examined by a veterinary surgeon prior to selection and quarantined for 2 weeks.

Animals received an opiate/midazolam combination sedative prior to surgical anesthesia. Anesthesia was induced with intravenous propofol and maintained with inhalational of isoflurane at 2–3% in 40% oxygen following the placement of a cuffed endo-tracheal tube. Animals were placed in lateral recumbency and the limb was shaved and aseptically prepared for surgery with chlorhexidine surgical scrub (5%), with a final wash with 70% (v/v) ethanol. The animals were draped with sterile drapes with surgical field exposed and an Ioban™ dressing applied.

A 10–15 cm parapatellar incision was made and the patella and patellar ligament exposed with blunt tissue dissection and hemostasis. The patella was then dislocated, laterally exposing the trochlear groove. Two defects, 6 mm in diameter and 6 mm in depth, were created in the central trochlear groove using sequentially larger drill bits. Care was taken not to damage the cartilage surrounding the defect. Thorough irrigation was used to remove any particulate matter from the defect and surrounding tissues. Sterile samples were manually press-fit into the defect site, flush with the surface of the joint.

Six replicates for each group were implanted into the 24 defects. The empty defects, MaioRegen™, and acellular scaffolds were paired randomly, while the chondrocyte seeded scaffolds were implanted together in pairs in three animals (Table S2). The segregation of the chondrocyte seeded scaffolds from the other conditions was performed to isolate the effect of any immune response to the allogeneic cell population and to mitigate the effect of chondrocyte migration through the synovial fluid to the neighboring defect site. The patella was then relocated and all internal tissue layers sequentially closed with absorbable sutures. The skin was closed with interrupted sutures following adequate hemostasis. The wound was then cleaned and dressed with an Ioban™ dressing.

Animals were recovered from general anesthesia and extubated on return of laryngeal reflexes. Pulse-oximetry, electrocardiography, and capnography were monitored throughout. Animals returned to a recovery area once conscious and stable and were given access to food and water once weight bearing and fully conscious. Animals were closely monitored for 72 h post-surgery, with wound inspection, appetite and lameness assessment as priorities. Postoperative analgesia was provided with fentanyl transdermal patches and oral NSAIDs, and adjusted according to individual requirements.

Animals were euthanized with intravenous administration of Lethabarb® at 6 months, the defect areas harvested, and gross pathology of the defect site was recorded. Samples were fixed in 4% formaldehyde at room temperature for 24 h. Fixed samples were cut in half, bisecting the scaffold perpendicular to the articulating surface to expose the full osteochondral gradient and integration with surrounding bone and cartilage. Samples were then transferred to 70% (v/v) ethanol and stored at 4 °C.

### 2.13 Micro-computed tomography

Samples were scanned with a micro-computed tomography (μCT) scanner (μCT 40, Scanco Medical, Brüttisellen, Switzerland) at a source voltage of 70 kV and a current of 114 μA. The samples were scanned in a tube with a diameter of 20 mm in 70% (v/v) ethanol. An isotropic voxel size of 20 μm was produced. μCT images were quantified for bone volume fraction using BoneJ [35], which is a plugin for ImageJ, and the SCANCO Medical μCT software suite, which is a proprietary μCT software package. In BoneJ, the workflow proceeded by loading the image sequence and binarizing with a threshold of 60%. The region of interest (ROI) was manually selected based on the original size of the injury and remaining irregularities (voids) in the subchondral bone. BoneJ was then used to calculate the Bone Volume (BV), Total Volume (TV), and Bone Volume Fraction (BV/TV). Using Scanco’s software, the ROI was manually selected using similar criteria. A lower threshold of 220 (minimum/maximum: 0/1000) was chosen to distinguish mineralized bone from background noise. The 3D reconstruction and mineralized content of each sample was then calculated with SCANCO’s proprietary algorithms to find BV, TV and BV/TV.

### 2.14 Histology

A subset of samples was embedded in Technovit 9100 NEU resin (Heraeus Kulzer, Wehrheim, Germany) without decalcification. Ground sections of the samples were obtained using an EXAKT ground sectioning system (Norderstedt, Germany) as previously described [36]. Briefly, thick sections (~200 μm) were cut using an EXAKT 310 diamond saw and ground and polished to 50 μm for with an EXAKT 400 CS micro-grinder for histological analysis. The ground sections were stained with the Goldner’s trichrome stain to visualize tissue morphology and mineralization.

A second subset of samples were demineralized in EDTA and embedded in polyester wax. Sections were cut at 5 μm and stained by either hematoxylin and eosin (H&E), picrosirius red and Alcian blue, or immunohistochemistry. Picrosirius red sections (counter stained with Alcian blue) were imaged in two different modes: bright field and polarized light. 3,3′-diaminobenzidine (DAB) immunohistochemistry was performed to identify collagen type I, collagen type II, and PRG4 localization and followed a previously published protocol for articular cartilage [37]. Due to method development and limited sections, only a subset of the total samples was able to be used for each histological stain.

Single blinded histological scoring of the repair sites was performed using a modified version of the ICRS II Histology Scoring System [38, 39]. A total of four graders were trained to evaluate each metric of interest. Matrix staining and tissue morphology were assessed on picrosirius red sections under bright field and polarized light, respectively. Cell morphology was assessed on H&E sections. Basal integration, surface architecture, subchondral bone, and overall histology were assessed on Goldner’s trichrome stained sections. Collagen type I (1310–01, Cambridge Biosciences), collagen type II (1320–01, Cambridge Biosciences), and PRG4 (MABT400, Sigma-Aldrich) localization were assessed using immunohistochemistry labeled sections.

### 2.15 Raman spectroscopy mapping and principal component analysis

A third subset of the samples was further analyzed using confocal Raman spectroscopy. Thick sections (20 μm) obtained by a heavy-duty sledge microtome (Reichert-Jung; International Medical Equipment) were adhered to CaF_2_ slides (Crystran) via a thin layer of gelatin. Sections were de-plasticized with 3 × 30 min washes in 100% acetone followed by rehydration in an ethanol-water dilution series (100, 90, 70, and 0%). Slides were fixed to the bottom of 100 mm diameter petri dishes, submerged in deionized water, and placed on a confocal Raman spectroscopic imaging system (Alpha 3000, WITec). A 532 nm wavelength laser at ~35 mW was focused on the sample surface through a 63X/1.0 NA Vis-IR water immersion objective (Zeiss). A 100 μm diameter fiber optic cable acting as a confocal pinhole was used to couple the microscope to a spectrograph (UHTS 300, WITec) with a 600 groove/ mm grating. Regions of interest (500 × 500 μm) were mapped at 10 μm intervals with a 0.7 s integration time. Spectral data was collected from 498 to 3673 cm^−1^ and background corrected using a shape factor of 250. The integrated intensity was normalized from 498 to 3673 cm^−1^.

Univariate analysis at 2939 ± 22 cm^−1^, a known Raman shift for lipids and proteins that corresponds to CH_3_ vibrations was used to visualize matrix deposition in the repair regions [40]. An unsupervised principal component analysis, performed on the fingerprint region of the Raman spectra (498–1800 cm^−1^), was then used to find the sources of greatest spectral variation between the repair sites.

### 2.16 Statistics

Data are reported as the mean ± 95% confidence interval unless stated otherwise. The number of replicates (technical and biological) are identified on each figure or within the text. All statistical tests were performed in SPSS®. Numerical data was checked for normality using the Shapiro-Wilk’s test and for homogeneity of the variances using Levene’s test.

If the data satisfied normality and homogeneity tests, a one-way ANOVA was used to detect significant effects. Multiple comparisons were performed using Tukey’s method. Welch’s ANOVA was conducted if normality was satisfied but not homogeneity of variances. If neither normality nor homogeneity of variances were satisfied then a Kruskal- Wallis H test was performed. Scored histological data was further evaluated for inter-rater (grader) bias using the difference method and tested against the null hypothesis that the difference between any two graders is equal to 0 [41]. Statistical significance was set at p < 0.05 for all tests.

## 3 Results and discussion

### 3.1 Biomimetic scaffold fabrication

The primary aim of this study was to produce a zonal microstructured scaffold and evaluate its efficacy in an osteochondral defect model. The proposed design built upon our previous work [9,27,28] and consisted of an aligned fiber surface, intermediate isotropic zone and a distinct deep zone with higher stiffness and vertical pore morphology. The primary novel attributes of this design compared to our previous work are the addition of a stiff deep zone, and as mentioned later, an osteointegrating region. We achieved this architecture by bonding a spherical porogen-leached zone (PLZ) and directionally frozen zone (DFZ) with electrospun fibrous layers on the upper and lower surfaces (Fig. 1A). During initial scaffold fabrication, we found a consistent issue with the integrity of the design. While the PLZ and DFZ produced a single continuous foam as per the design, the electrospun fibers were weakly integrated and delaminated easily from the porous foams. This was an issue we began to address in 2014 with our bilayered scaffolds [20]. To overcome the weak interfacial bond, we deposited the first few layers of fibers at 50 mm from the foam surface, 50% closer than the standard tip-to-target distance of 100 mm. At this distance the electrospun fibers had a higher residual solvent content, resulting in increased fiber-fiber (Fig. 1B) and fiber-foam adhesion (Fig. 1C–E). This modification led to a reproducible strategy for creating a fully integrated microstructured scaffold. We confirmed the mechanical integrity of the bond fiber-foam bond by manually pulling on the fibrous layer with tweezers.

The porosity of the microstructured scaffolds were 97 ± 1%. The median major axis pore size in the X–Y plane was 2.1, 123.3, and 44.4 μm for the fibrous zone, PLZ, and DFZ, respectively. It should be noted that the pore geometries in the DFZ and fibrous zones are anisotropic, with smaller pores in the orthogonal direction. We verified axial interconnectivity between the PLZ and DFZ by seeding chondrocytes in the PLZ and later verifying their presence in the DFZ (we did not include the fibrous zones for this validation).

### 3.2 Scaffold mechanics and strain partitioning

**Fig. 2:**
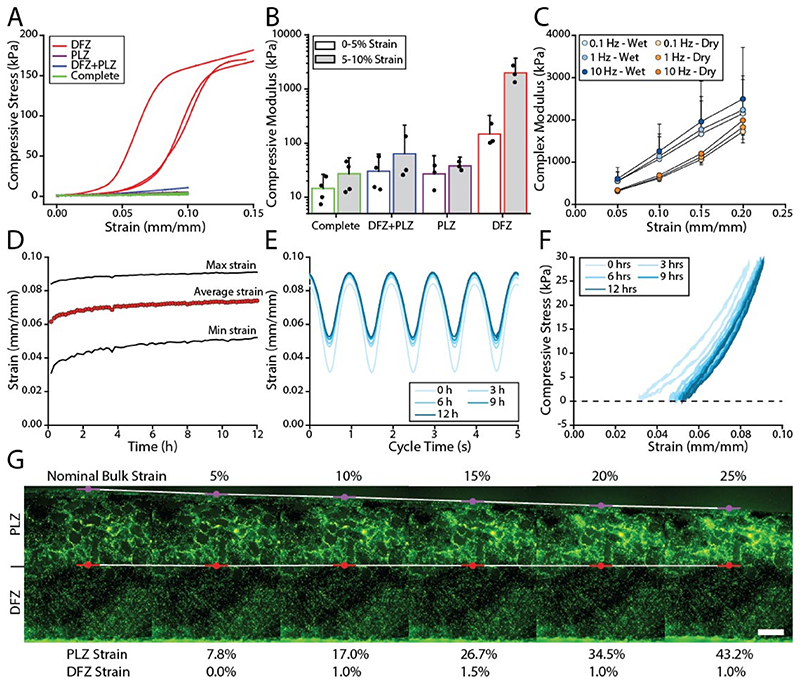
Mechanical performance of zonal microstructured scaffolds. (A) Unconfined compressive stress versus strain for the DFZ, PLZ, DFZ + PLZ, and complete scaffolds. Compressive force and displacement are shown in Figure S2. (B) The calculated compressive modulus (0–5% and 5–10% strain) for each level of scaffold fabrication. Error bars indicate the 95% confidence interval for N = 3 to 4 samples. Note that the data is plotted on a log scaled vertical axis and only positive error bars are shown. (C) The complex modulus is shown as a function of strain (5, 10, 15, and 20%) for different frequencies (0.1, 1, and 10 Hz) and hydration conditions (dry and wet). Error bars indicate the 95% confidence interval for N = 3 samples. For figure clarity, the dry samples are shown with negative error and wet samples with positive error. (D) The mean, maximum, and minimum strain for a 12 h cyclic compression test at 1 Hz under a 28 kPa stress amplitude. Stress amplitude is shown in Figure S3. (E) The strain profile is plotted every 3 h during the 12 h test. (F) Compressive stress is plotted against strain to indicate compressive hysteresis. (G) A fluorescently labeled scaffold (PLZ + DFZ) was compressed from 0 to 25% strain and demonstrates strain partitioning between the zones. Scale bar = 250 μm.

One rationale for the incorporation of a directionally frozen zone in the optimized scaffold design was to generate scaffolds with substantially increased compressive stiffness in the deep zone; thereby mimicking the mechanical gradients across the depth of articular cartilage [42–45]. The isolated DFZ proved to be stiffer (1974 kPa) than the PLZ (38 kPa), as shown in Fig. 2A–B. When the two foams were bonded together the apparent modulus was 64 kPa, which is a modest increase over the PLZ but a substantial reduction compared to the DFZ. To verify the zonal properties remained intact, the zonal strain was tracked as a function of macroscale strain (Fig. 2G). We observed that nearly 98% of the strain accumulated in the PLZ demonstrating a substantially stiffer DFZ and the retention of zonal mechanics. The complete scaffold, which included an electrospun surface, had an apparent modulus of 27 kPa. The increased compliance at the surface of the microstructured scaffold is similar to native articular cartilage, which undergoes an 8-fold reduction in compressive modulus compared to the deep zone [46]. It should be noted that overall the compressive modulus of the microstructured scaffold was substantially lower than native articular cartilage which is typically on the order of 10 MPa [47–49].

In addition to the zone dependent mechanics the scaffolds also displayed strain stiffening and rate dependent behavior (Fig. 2C). The strain stiffening effect is a common feature of cellular structures undergoing compressive deformation [50] and is also observed in articular cartilage [42]. The effect of cycling frequency on the compressive modulus suggests a viscoelastic mechanism due to polymer chain relaxation. Interestingly, when testing submerged specimens subjected to the same cycling conditions, they showed increased stiffness and greater frequency dependence. This shift in performance may be explained by a further increase in viscoelastic and/or poroelastic effects [51].

We evaluated the mechanical stability of the scaffolds under cyclic loading by imposing a 28 kPa stress amplitude at 1 Hz for 12 h. In Fig. 2D, the mean strain rapidly increased over the first 30 min; however, beyond 3 h the mean strain only increased by 0.044 ± 0.002% strain/h, showing steady state performance. In Fig. 2F, the area bounded by the stress-strain curves shows hysteresis between the loading and unloading cycles and further supports a viscoelastic mechanism.

### 3.3 Zonal microenvironments support in vitro tissue engineering

We seeded full-thickness scaffolds by direct injection of a 50 μL bolus of cell suspension containing 10 × 10^6^ cells. First, we injected 45 μL into the gelatin-leached part of the scaffold and then deposited the remaining 5 μL on the superficial electrospun surface. Preliminary histological evaluation of scaffolds showed near-complete cell and tissue infiltration at 8 weeks *in vitro* (Figure S4).

**Fig. 3:**
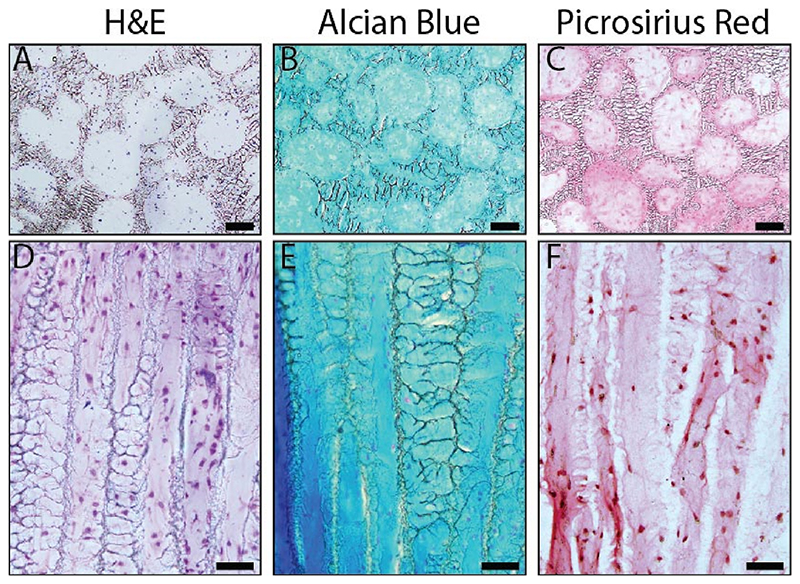
Histological analysis of tissue formed within the scaffold, seeded with 10 × 10^6^ bovine chondrocytes, and cultured *in vitro* under chondrogenic conditions for 4 weeks. The PLZ (A-C; scale bars = 100 μm) and DFZ (D-F; scale bars = 50 μm) were stained with (A,D) hematoxylin and eosin (H&E) for nuclear morphology and non-specific tissue visualization, (B,E) Alcian blue for GAG, and (C,F) picrosirius red for collagen deposition. (For interpretation of the references to color in this figure legend, the reader is referred to the Web version of this article.)

We performed histology (H&E, Alcian blue, and picrosirius red) after 4 weeks of *in vitro* tissue engineering to characterize the chondrogenic potential of these scaffolds (Fig. 3). We found ECM throughout the scaffold porosity in both the PLZ and DFZ. Alcian blue staining showed a uniform distribution of glycosaminoglycan (GAG) throughout both zones, and picrosirius red staining showed greater collagen density on the surface of pores. In addition, we used collagen type II fluorescent immunohistochemistry and second harmonic generation imaging on a representative scaffold (Figure S5), which showed evidence of a developing cartilage-like matrix.

DNA and sGAG content of the microstructured scaffold were biochemically evaluated (Figure S6). Results showed increased sGAG production over time with higher levels in the PLZ and DFZ compared to the upper and lower fiber zones. Upon normalizing the sGAG content to DNA content it appeared that the increased sGAG production in the PLZ and DFZ was due to a greater cell content. Real-time quantitative PCR confirmed the beneficial effects of cell density, with increased *COL2A1, AGC*, and *SOX9* expression at 10 × 10^6^ cells compared 1 × 10^6^ cells (Figure S7). In preliminary work we also investigated 300–500 μm diameter porogens and found lower gene expression for key chondrogenic markers compared to the 100–300 μm diameter porogens used in this work.

### 3.4 Assessment in skeletally mature porcine osteochondral defect model

We next evaluated the scaffolds in a skeletally mature porcine osteochondral defect model. For this, we thermally bonded the microstructured scaffolds to a bone mimic to provide a zone for osteointe- gration. For the bone mimic, we used melt-electrowriting to fabricate an open-pore PCL lattice. The final scaffold yielded a 6 mm diameter by 6 mm thick osteochondral implant.

In 12 animals, we created 24 osteochondral defects that were repaired with one of four treatments (six replicates per treatment group): acellular microstructured scaffold, cell-seeded microstructured scaffold, MaioRegen™ scaffold, or empty defect (Figure S8 and Table S2). Animals were euthanized at 6 months and the defect areas were harvested for analysis.

**Fig. 4:**
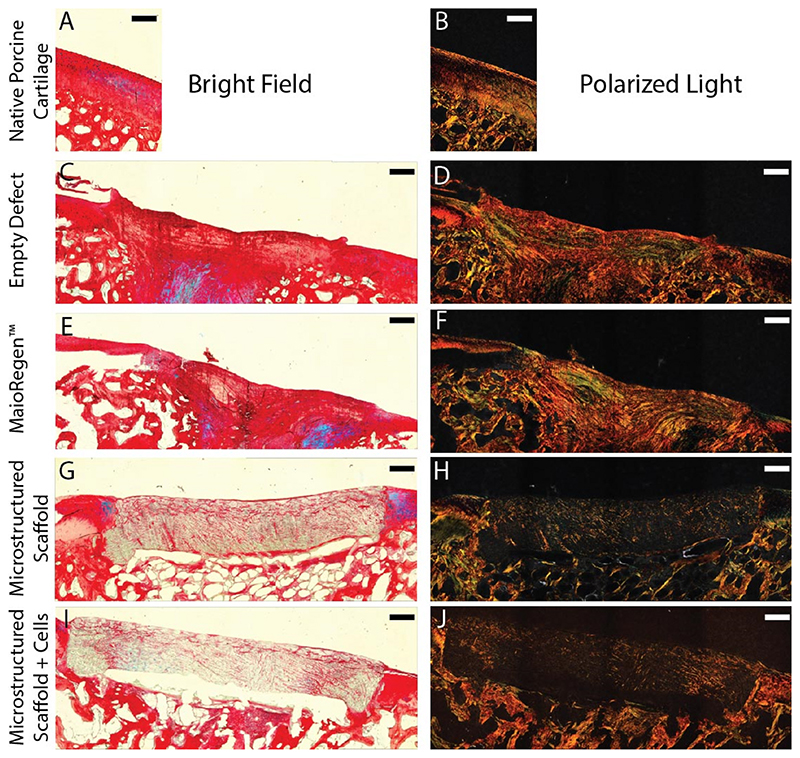
Optical microscopy of picrosirius red and Alcian blue stained sections of the defect site after 6 months. (A,C,E,G,I) Bright field images of the stained sections demonstrate the spatial composition of the repair sites. (B,D,F,H,J) Polarized light was used to detect collagen alignment due to its orientation dependent refraction (birefringence). Scale bars = 500 μm. (For interpretation of the references to color in this figure legend, the reader is referred to the Web version of this article.)

Macroscopic evaluation of the joint surfaces at 6 months revealed integrated scaffolds and tissue for each group. We qualitatively assessed cell ingress and matrix, collagen, and glycosaminoglycan deposition using H&E, picrosirius red, and Alcian blue, respectively (Fig. 4 and S9). We saw clear evidence of matrix deposition throughout the microstructured scaffolds (acellular and cell-seeded); however, the retention and minimal degradation of these implants at 6 months may have contributed to the limited matrix deposition compared to the controls (empty and MaioRegen™). Interestingly, we did not find differences between the acellular and cell-seeded microstructured scaffolds, suggesting that pre-seeding may provide no additional benefit. Using polarized light microscopy, we observed poor collagen alignment in the controls, while collagen deposition in the microstructured scaffolds was insufficient to visualize overall alignment. It should be noted that MaioRegen™ is composed of equine collagen type I and the observed defect filling may not be entirely the result of new matrix production.

**Fig. 5:**
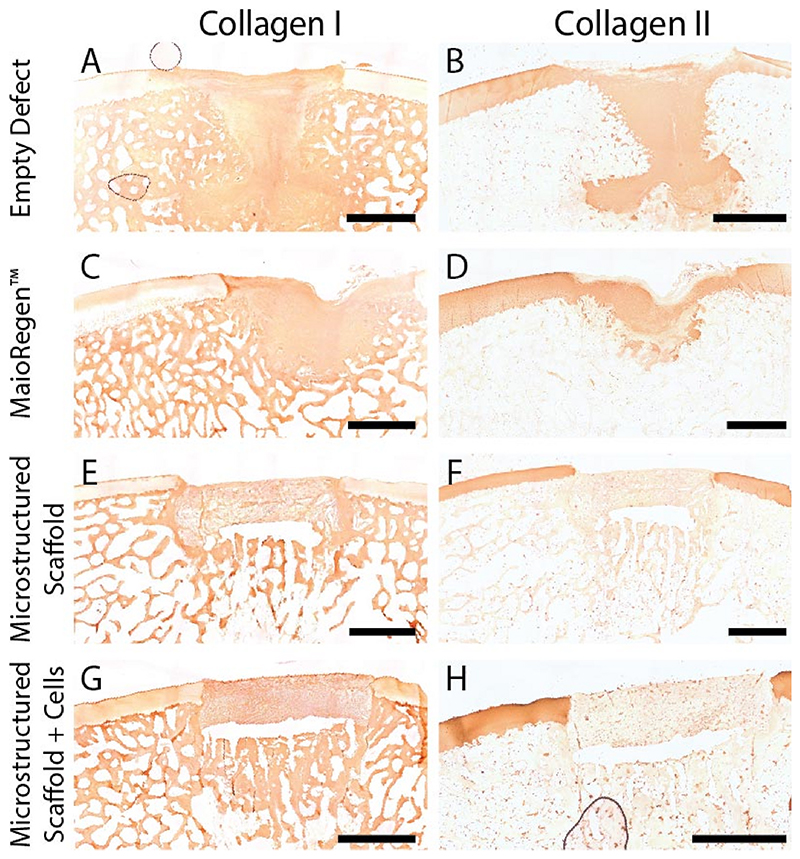
Collagen type I (A,C,E,G) and type II (B,D,F,H) localization using DAB immunohistochemistry. Representative paired sections for each repair type are shown. To improve visualization, collagen type I images were contrast enhanced in ImageJ (100–255) and white balanced using the plugin BIOP Simple Color Balance. Scale bars = 2 mm. (For interpretation of the references to color in this figure legend, the reader is referred to the Web version of this article.)

Articular cartilage is predominantly composed of collagen type II while bone is largely collagen type I. We used 3,3′-diaminobenzidine (DAB) immunohistochemistry to identify collagen type and localization (Fig. 5). We observed collagen type I in both the subchondral bone and repair tissue. The presence of collagen type I in the repair site is consistent with the formation of fibrocartilage, which is generally seen as an inferior tissue [52]. When staining for collagen type II, we found clear evidence in the articular cartilage surrounding the defects. In addition, we saw collagen type II in the repair sites for the empty defect and MaioRegen™ controls. The microstructured scaffolds (with and without allogeneic chondrocytes) did not show evidence of collagen type II production in the repair site. Interestingly, we found PRG4, which has been identified as a key boundary lubricant for articular cartilage [53], at both the sliding surface and within the defect for all treatment conditions (Figure S10). While the presence of PRG4 is thought to be indicative of articular cartilage and promote a low friction environment, it did not mirror the depth dependent gradient normal cartilage is known for.

**Fig. 6:**
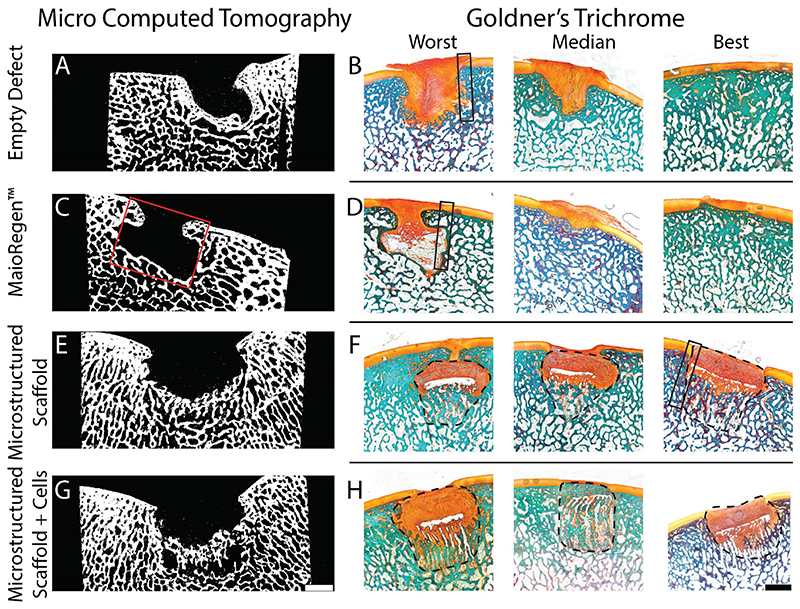
(A,C,E,G) Representative μCT projections and (B,D,F,H) Goldner’s trichrome stained sections of the repair site at 6 months. Goldner’s trichrome stains mineralized tissue blue/green and collagen orange [54]. The Worst, Median, and Best repair was qualitatively determined for each treatment type. The red box (C) indicates the approximate size and location of the original defect. The original defect was 6 × 6 mm (depth x diameter); however, to account for the approximate cartilage thickness (1 mm) a 5 × 6 mm box is drawn. Dashed lines are used to identify the microstructured scaffold margins at 6 months post implantation. Boxed regions are the reference locations for Figure S13. Images were background subtracted in ImageJ using the rolling ball method (radius = 20 pixels). Scale bars = 2 mm (scale bars apply to all images in the series). (For interpretation of the references to color in this figure legend, the reader is referred to the Web version of this article.)

We were able to visualize osteointegration and quantify bone volume fraction (BV/TV) using μCT. To perform the quantitative analysis, we used two different software packages (SCANCO Medical microCT software suite and BoneJ) (Figure S11). The reference values for normal subchondral bone BV/TV were 0.43 from Scanco and 0.67 from BoneJ (Figure S12). We detected significant effects of treatment on BV/TV using a one-way ANOVA. Post-hoc comparisons did not reveal significant differences between normal subchondral bone and the empty defect control while all other conditions had a significantly lower BV/TV. Preseeding the microstructured scaffolds with porcine chondrocytes did not have a significant effect on bone formation at 6 months. Representative μCT cross-sections are shown in Fig. 6 and demonstrate bone formation in the osteo aspect of the microstructured scaffolds.

Goldner’s trichrome stain was used to simultaneously visualize and differentiate mineralized tissue from nonmineralized collagen (Fig. 6) [54]. In all conditions new collagenous tissue filled the defect with qualitatively more tissue deposition in the empty and MaioRegen™ treated defects. The difference in new matrix deposition is believed to be a combination of the mechanical support provided by the visually intact microstructured scaffolds (i.e., less matrix deposition required) and the volume displaced by the implant. We observed new bone formation in the subchondral repair site for all conditions and there was clear evidence of osteointegration (Figure S13) for the microstructured scaffolds. The boundary between the host and repair tissue at the chondral interface could be easily identified due to differences in tissue morphology and was most apparent in the microstructured scaffolds. Despite the difference in tissue morphology, we observed a continuous interface for all conditions (Figures S13 and S14). In both the empty and MaioRegen™ treated defects there were several instances of ectopic growth (3 empty and 1 MaioRegen™) and fibrous pit formation (3 empty and 2 MaioRegen™), while one of the microstructured scaffolds experienced graft subsidence greater than 1 mm [55]. Graft subsidence for PCL scaffolds has been observed before and maybe attributed to a biomechanical mis-match between the implant and the trochlear groove [56].

**Fig. 7:**
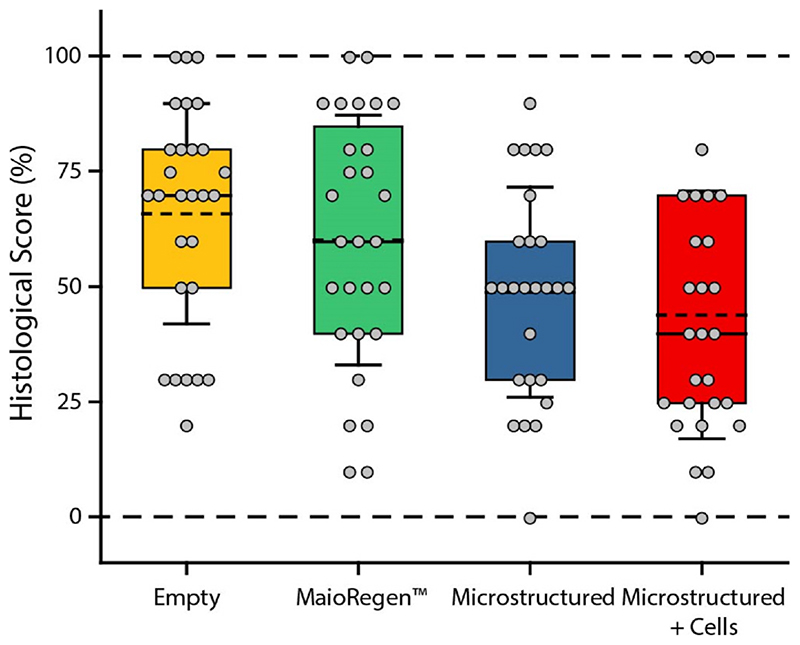
Histological scores for each treatment group from a single representative grader. The mean (dashed line), median (solid line), 25th to 75th percentile (box), and 1 standard deviation of the mean (whiskers) are shown. The data is provided in this format for visualization and generalization of the repair conditions. Statistical tests were not run on the aggregate data.

Four blinded graders were trained to use a modified version of the ICRS II Histology Scoring System [38]. Each grader ranked the histological sections on a scale of 0–100, with 100 being representative of native porcine cartilage. Graders scored the sections on: (1) matrix staining, (2) tissue morphology, (3) cell morphology, (4) collagen type I, (5) collagen type II, (6), lubricin, (7) basal integration, (8) surface architecture, (9) subchondral bone, and (10) overall histological appearance. The scored histological data did not meet the requirements for parametric testing; therefore, we used a Kruskal-Wallis H test to detect significant effects of treatment. Of the 10 metrics, we did not detect significant effects of treatment. We observed significant inter-rater bias for basal integration and surface architecture. Fig. 7 shows the aggregate scoring for one representative grader. It is clear from the aggregate data that there is an overall reduction in the histological score for the microstructured scaffolds. We propose that the retention and limited biodegradation of the scaffold at 6 months obstructs matrix deposition and is a key factor in the lower histological score. At longer time points the scaffold will undergo further biodegradation and may give way to increased matrix deposition.

**Fig. 8:**
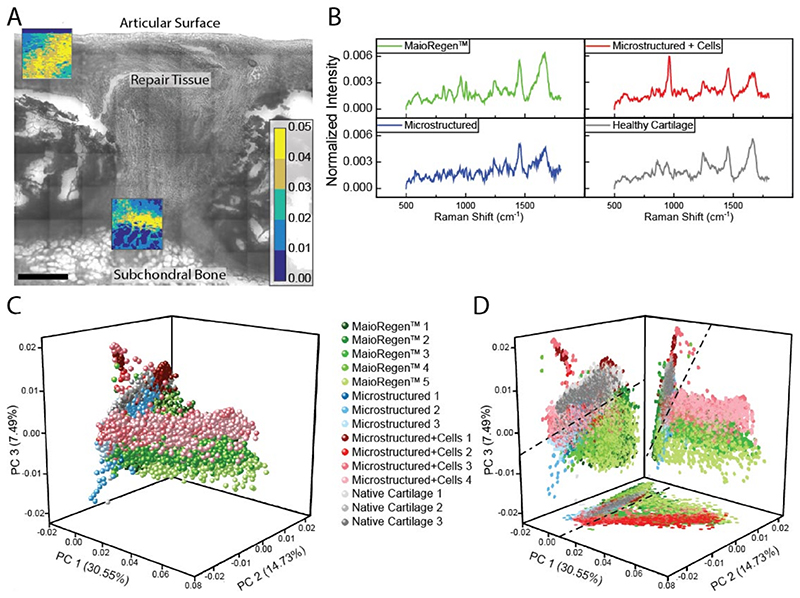
Raman spectroscopic imaging and principal component analysis of repair tissue. (A) Brightfield image of a defect treated with MaioRegen™. The brightfield image is overlaid with a univariate analysis of the Raman spectra (warmer colors = higher signal). Scale bar = 500 μm. (B) Mean spectra for each repair type. (C–D) Principal component analysis of Raman spectra. Marker color indicates repair type: MaioRegen™ (green), microstructured (blue), microstructured + cells (red), and healthy cartilage (grey). Variations in color intensity within groups correspond to different regions within the defect. (D) Dashed lines are used to identify the boundaries of healthy cartilage. (For interpretation of the references to color in this figure legend, the reader is referred to the Web version of this article.)

Raman spectroscopy, a label-free vibrational light scattering technique, was used to identify differences between healthy and repair tissues [57–59]. Univariate intensity mapping of the spectral peak at 2939 cm^−1^ (Figure S15), which corresponds to CH_3_ vibrations common across proteins and lipids, enabled identification of biological tissue that aligned with the corresponding bright field and histological images (Fig. 8A and S16). We then applied principal component analysis to the Raman spectra to identify the primary sources of spectral variation across different tissue regions and repair conditions. The first three principal components (PCs) (Figure S17) accounted for 52.75% of the observed spectral variance, with the spectrum of PC1 indicating hydroxyapatite (30.55%), PC2 representing collagen (14.7%), and PC3 containing a mixture of collagen and other ECM signatures (7.5%) [14, 59]. The PC scores in Fig. 8C and D suggest a low PC1 and high PC3 score are indicative of healthy cartilage. It seems reasonable that hydroxyapatite (PC1), a key mineral found in bone, is a negative marker for cartilage tissue quality, and that ECM is indicative of healthy cartilage. It is interesting that PC2, which represents collagen, is not a distinguishing feature for healthy cartilage; however, the collagen content and structure (alignment and fiber size) vary with depth in normal cartilage [14, 60] and the variation seen here may be a reflection of that. While we were not able to clearly identify PC3, likely due to contributions from multiple ECM components, it did suggest a biomolecular difference between the repair conditions and may serve as a marker for evaluating repair tissue quality. However, Raman spectroscopy mapping was only performed on a single representative sample from each group and thus a larger study involving multiple samples and possibly species will be needed to determine its significance. Furthermore, since Raman spectroscopy does not rely on biological stains it would be interesting to perform this type of analysis on unfixed tissue.

The design of the microstructured scaffold builds upon our prior work [9,27,28] and an extensive literature on the use of nanofibrous polymers, porogen leached foams, directionally frozen foams, and melt-electrowritten lattices for cartilage and bone repair [61–64]. Despite this, the microstructured scaffold showed a reduced histological score at 6 months in an osteochondral defect model. Our choice of biomaterial is likely a primary driver of the lower histological score. We selected PCL for this study as it is compatible with multiple fabrication technologies, has good bulk mechanical properties (0.4 GPa modulus and high ductility), is biocompatible, and degrades slowly. While the slow biodegradation was intended to provide prolonged microstructural cues to the developing cartilage matrix it appears to be one of the factors behind the limited matrix deposition. In future work, it would be worth considering a more rapidly degrading material such as poly (lactic acid) or poly (glycolic acid). Ultimately, we need a longer preclinical time point to fully evaluate the efficacy or lack thereof for the microstructured implant.

In moving towards clinical application, we will establish GMP production and further *in vitro* and *in vivo* testing to apply for an investigational device exemption (IDE) to support a clinical study on safety and efficacy of the acellular microstructured implant. The zonal microstructured scaffold would likely be classified as a non-degradable (degradation >30 days) and implantable Class 3 Medical Device, which would require premarket approval (PMA) prior to clinical use.

## 4 Conclusion

We combined different fabrication techniques to produce a zonal microstructured scaffold to better replicate the intricate structure of articular cartilage. The microstructured scaffold showed a nearly 100-fold change in modulus through its thickness and supported *in vitro* tissue engineering of chondrocytes out to 28 days with the production of collagen and glycosaminoglycan. Despite having a modulus much lower than native articular cartilage, the microstructured scaffold remained intact at 6 months in a large animal model of osteochondral defect repair. Furthermore, the microstructured scaffold experienced matrix deposition, osteointegration, and maintained a flush articular surface. Principal component analysis performed on Raman spectral images of the different tissues identified variations in both the content and type of ECM produced in the repair sites. While the evidence at 6 months does not suggest a superior repair mechanism for the microstructured scaffold, it does demonstrate a robust and stable repair that may offer advantages at longer time points.

## Supplementary Material

Supplementary Materials
