# Supplementary material for: In vitro and in vivo investigation of a zonal microstructured scaffold for osteochondral defect repair
Source: Biomaterials. Author manuscript; Available in PMC 2024 Jan 5. (PMC7615488; doi:10.1016/j.biomaterials.2022.121548)
Supplement: Supplementary Materials [file EMS175896-supplement-Supplementary_Materials.doc]

***In vitro* and *in vivo* investigation of a zonal microstructured scaffold for osteochondral defect repair**

Steele, Joseph A M ^1,2,3,4§^

Moore, Axel C ^1,2,3§^

St-Pierre, Jean-Philippe ^1,2,3^

McCullen, Seth D ^1,2,3^

Gormley, Adam J ^1,2,3,4^

Horgan, Conor C^1,2,3^

Black, Cameron RM ^5,6,7^

Meinert, Christoph ^6+^

Klein, Travis ^6,8^

Saifzadeh, Siamak ^7^

Steck, Roland ^7^

Ren, Jiongyu ^6,8^

Woodruff, Maria A ^6,8*^

Stevens, Molly M ^1,2,3,4*^

**S1.0 Supplemental Methods**

**S1.1 Scaffold Production**

The composite scaffolds were produced in a step-wise manner, first generating a porogen-leached/directionally-frozen integrated foam then electrospinning fibers onto the top and bottom of the scaffold (**Figure S1**). Prior to scaffold fabrication, thermoset gelatin microspheres were produced by an optimized protocol.


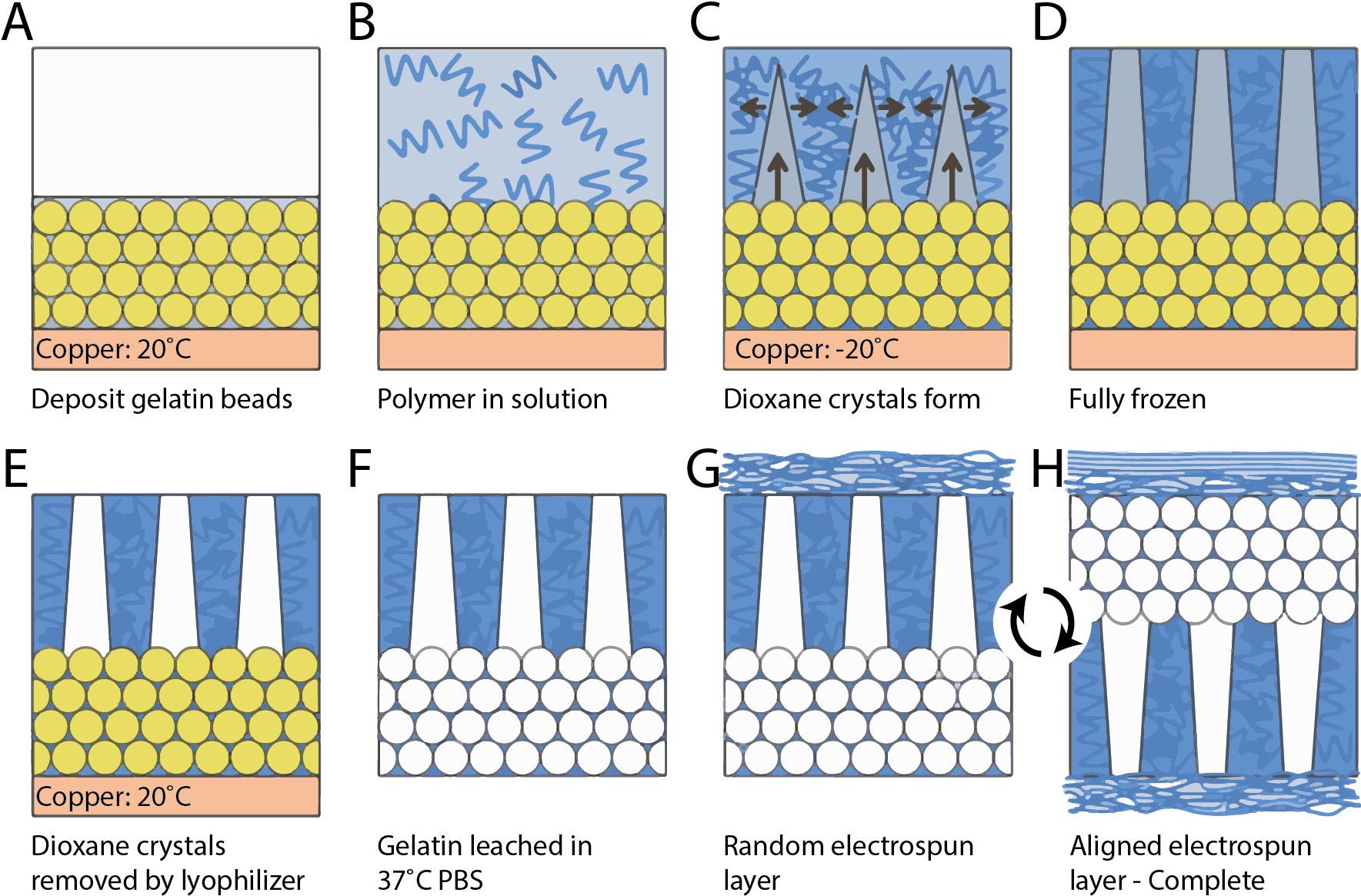


Figure S1. Scaffold production process. (A) Gelatin microspheres in 8% (w/v) PCL-dioxane were deposited on a copper sheet and dried. (B) A 12% (w/v) PCL-dioxane solution was added to the particles and directionally frozen (C and D). Dioxane was removed by lyophilization (E) and gelatin was removed by incubation in warm water followed by PBS (F). The directionally frozen surface was electrospun with random PCL fibers (G) and the porogen leached surface was electrospun with random-to-aligned PCL fibers (H).

**S1.2 Gelatin Microsphere Production**

The production of porogen-leached foams used uncrosslinked thermoset gelatin microspheres, as they can be prepared as populations of homogenous sizes and produce spherical pores, as opposed to crystalline rectilinear salt crystals. Gelatin microspheres were produced by an oil-water emulsion protocol developed and optimized for this scaffold.

A 20% (w/v) solution of porcine skin type A gelatin in distilled water at 60°C was added in a drop-wise manner to a 500 ml Schott bottle containing 250 ml sunflower oil (Waitrose, UK) and 0.5% (v/v) Tween 20. Throughout, the system was mixed at 500 rpm with an upright laboratory mixer (Eurostar digital, IKA, UK). The oil/water emulsion was allowed to stabilize for 10 min, after which the mixture was cooled by jacketing with ice water to thermoset the gelatin. Once the gelatin was set, a volume of -20°C acetone equal to the volume of oil was added and mixed overnight at 4°C to dehydrate the particles and cut the viscosity of the oil. The acetone/oil/water mixture was decanted and the particles washed with acetone at 4°C for 48 h with 2 changes of acetone. Particles between 100 and 300 µm diameter were collected using graded stainless steel meshes, dried under vacuum, and stored in sealed containers until required.

**S1.3 Scaffold Production Protocol**

A slurry of 5 g of gelatin microspheres suspended in 7 mL of 8% (w/v) PCL-dioxane was deposited onto a clean 450 μm thick copper plate to a thickness of 1 mm within an 80 x 80 x 2 mm thick PDMS mold and allowed to dry overnight (**Figure S1A**). Additional 8% (w/v) PCL in dioxane was deposited onto the dried gelatin microspheres to a final depth of 2 mm (**Figure S1B**). The samples were then directionally frozen by placing the copper sheet on a -20°C cold-plate (PolarBear Plus, Cambridge Reactor Design Ltd.). The PCL/dioxane froze from the gelatin interface toward the free surface and concentrated the PCL at the crystal boundaries (**Figure S1C**). The cold-plate mimicked the control and rates obtained by the traditional cold-finger approach to directional freezing, but sacrificed some thermal homogeneity in exchange for the large size of scaffolds produced (80 x 80 mm). When fully frozen, the columnar dioxane crystals were removed by lyophilization, leaving open columnar channels formed by the partitioned PCL (**Figures S1D** and **S1E**). The gelatin microspheres were removed from the scaffolds by washing in warm water (37°C) and PBS, and finally dried at 40°C (**Figure S1F**).

A layer of randomly-oriented fibers was electrospun onto the directionally-frozen surface using a custom electrospinning apparatus. Foams were attached to an electrically grounded voltage-driven rotating mandrel 100 mm wide and 200 mm diameter, rotating at 100 rpm to collect random fibers. A solution of 12% (w/v) PCL in 1,1,1,3,3,3-hexafluoroisopropanol (HFIP), was spun onto the foam through a 19G blunt needle at 2 mL/h using a programmable syringe pump (Kd Scientific Model KDS 100 CE, Sandbach, Cheshire, UK) with an applied 11 kV potential. The initial distance between the foam and the needle was 50 mm, increased after 1 h to the standard 100 mm for a further 7 h. The scaffold was then flipped and a second layer of fibers was electrospun onto the opposite particle-leached surface, transitioning from adhesive random (1 h, 50 mm, 100 rpm) to standard random (1 h, 100 mm, 100 rpm) to aligned deposition (6 h, 100 mm, 2000 rpm) (**Figure S1H**).

**S1.4 RT-qPCR Primers**

Table S1. RT-qPCR primers for the assessment of cartilage-specific gene expression in bovine chondrocytes.

| **Gene** | **Forward** | **Reverse** | **Efficiency** |
| --- | --- | --- | --- |
| COL2A1 | CCACTGCAAGAACAGCATTG | CCAGTTCAGGTCTCTTAGAG | 60°C – 98% |
| COL1A1 | CATTAGGGGTCACAATGGTC | TGGAGTTCCATTTTCACCAG | 60°C – 92% |
| AGC | CACTGTTACCGCCACTTCCC | GACATCGTTCCACTCGCCCT | 60°C – 93% |
| SOX9 | AACGCCGAGCTCAGCAAG | ACGAACGGCCGCTTCTC | 62°C – 96% |
| 18S | GTAACCCGTTGAACC | CCATCCAATCGGTAGTAGTAGCG | 60°C – 104% |

**S2.0 Supplemental Data**

**Figure S2.** Force-displacement data for the DFZ, PLZ, DFZ+PLZ, and complete scaffolds.

Figure S3. Compressive stress is plotted for 5 cycles (1 Hz) every 3 h for a 12 h cyclic compression test.

Figure S4. Scaffold cross-section at 8 weeks showing tissue and cell infiltration. (A) H&E staining. (B and C) Alcian blue staining. (C) A higher-magnification image of the DFZ+PLZ boundary. Scale bars are 250, 250, and 50 µm for A, B, and C respectively.


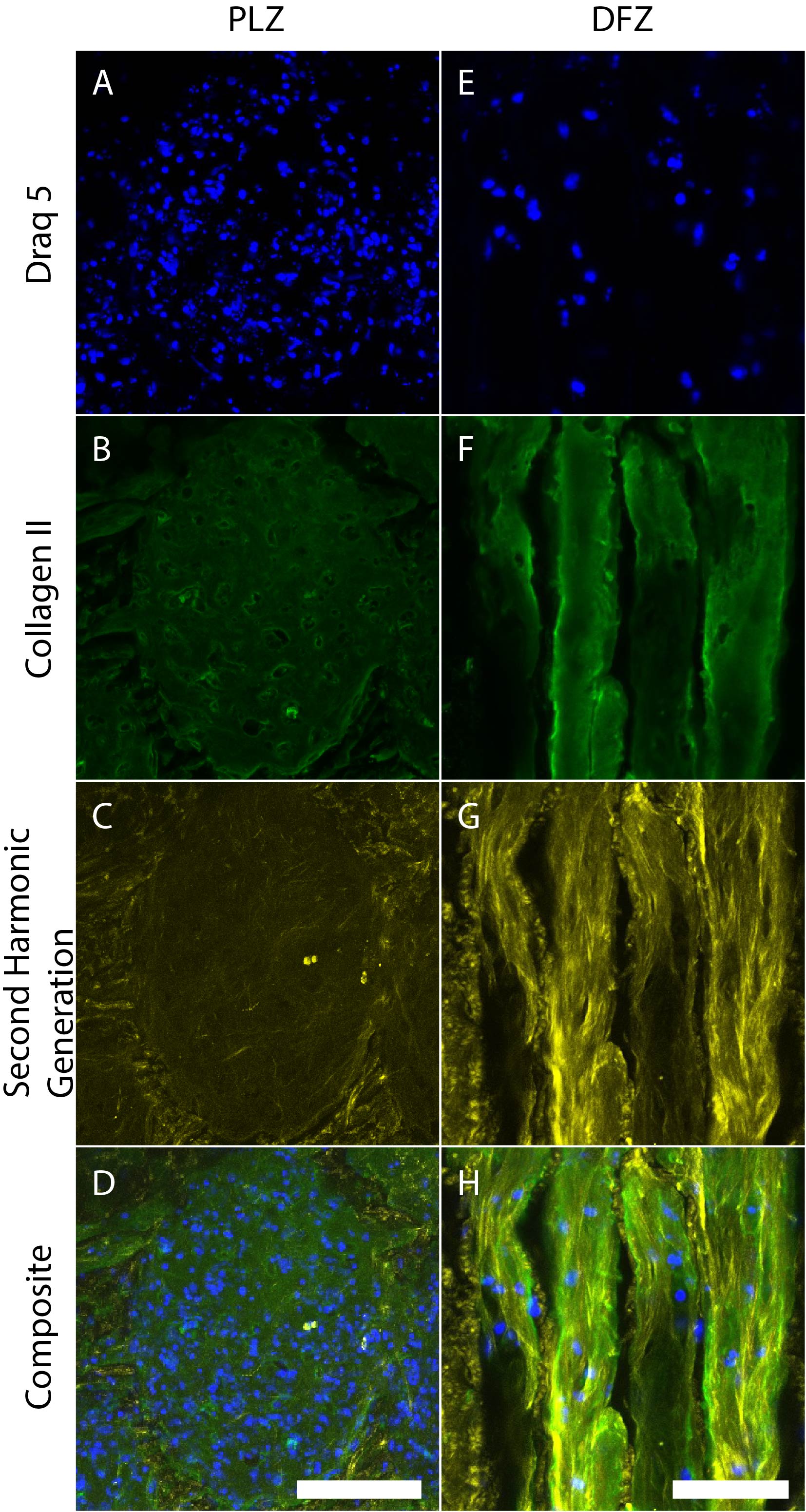


**Figure S5.** An *in vitro* tissue engineered scaffold was stained for cell nuclei (A,E) and collagen type II (B,F), and imaged using second harmonic generation (C,G) in the PLZ (A-D) and DFZ (E-H). Scale bars = 100 μm (D) and 50 μm (H) and apply to all images in A-D and E-H respectively.

 
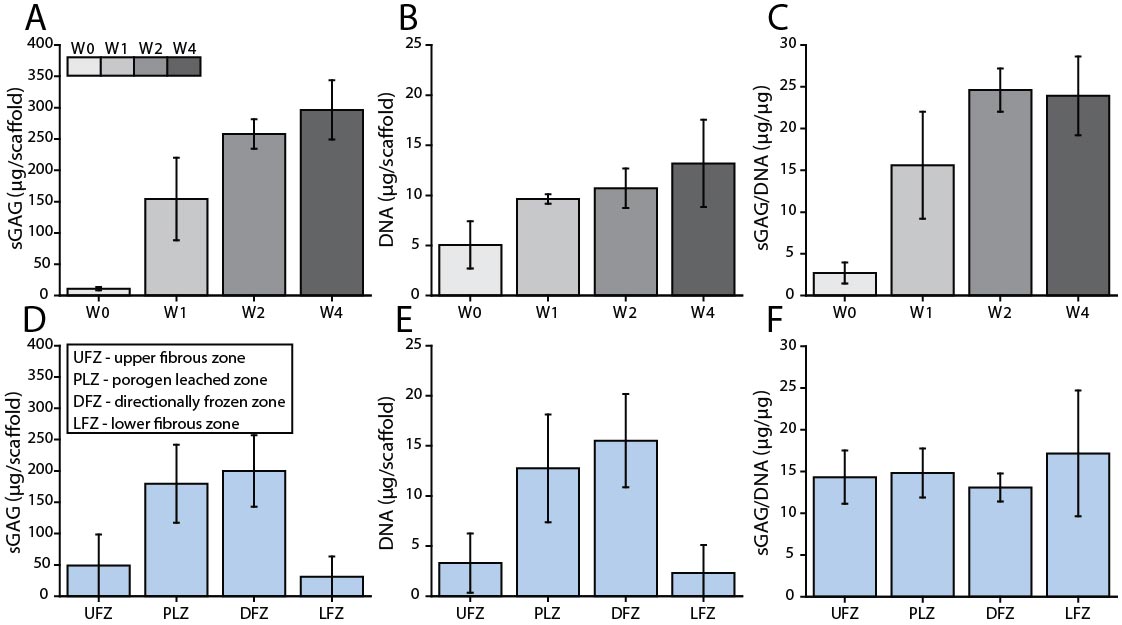


Figure S6. Temporal (A,B,C) and spatial (D,E,F) biochemical assessment of *in vitro* tissue engineered scaffolds. Temporal changes were quantified for the complete scaffold at weeks 0, 1, 2, and 4. Spatial (upper fibrous zone, porogen leached zone, directionally frozen zone, and lower fibrous zone) changes were quantified after 4 weeks of *in vitro* culture. The sGAG (A,D), DNA (B,E) and sGAG/DNA ratio (C,F) were measured and calculated. Error bars represent the 95% confidence interval for N = 3-4 samples with technical triplicates for all samples.


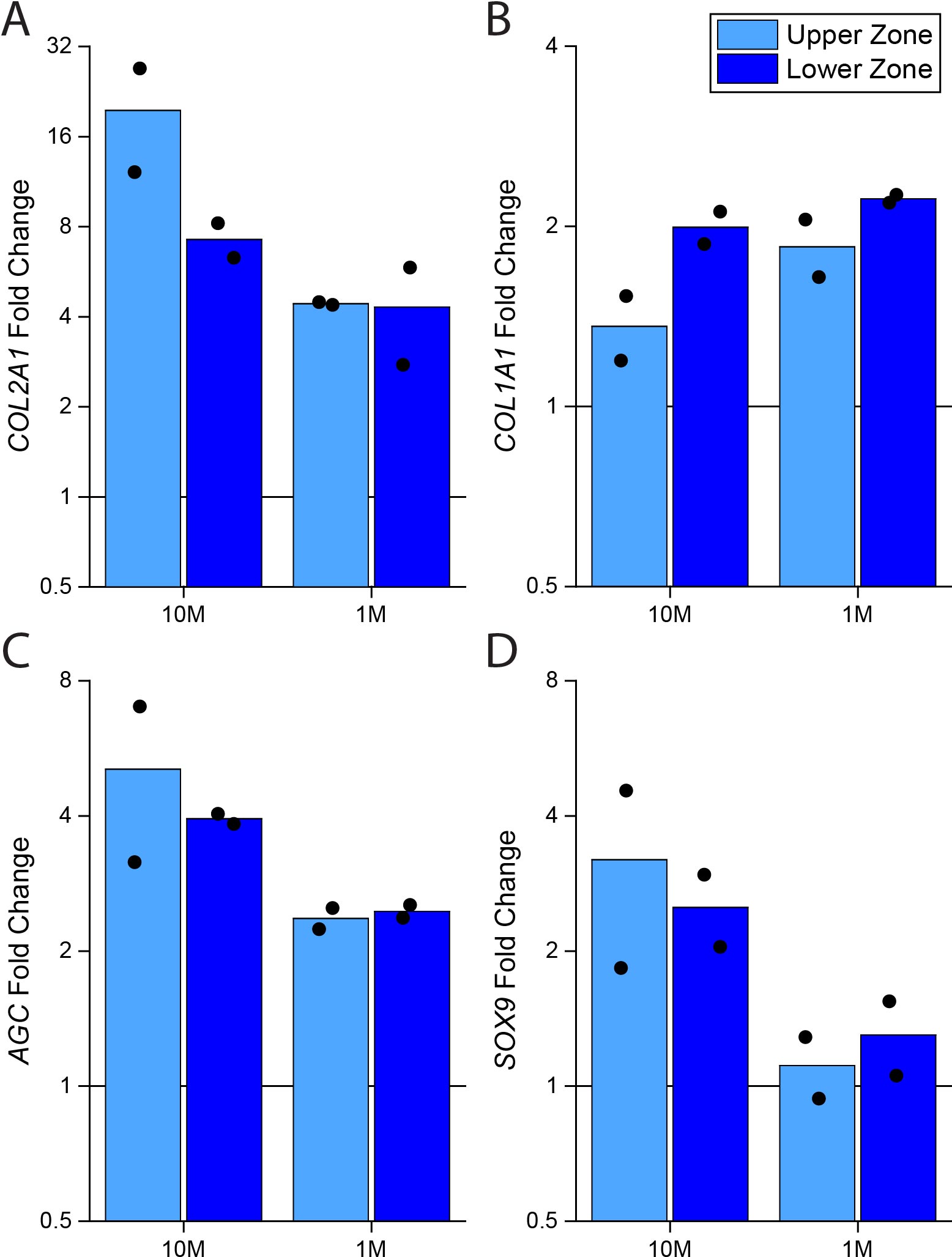


Figure S7. Gene expression profiles for scaffolds with 10 x 10^6^ (10M) or 1 x 10^6^ (1M) passage 2 bovine chondrocytes following 7 days of *in vitro* culture in chondrogenic medium. For a semi-zonal analysis, the scaffolds were subdivided into two halves at the PLZ-DFZ boundary. (A) Type II collagen (*COL2A1*), (B) type I collagen (*COL1A1*), (C) aggrecan (*AGC*), and (D) *SOX9* are expressed as fold changes relative to day 0 controls and normalized to the 18S housekeeping gene. Note that the vertical axis range is not uniform between genes and it is shown as log base 2.


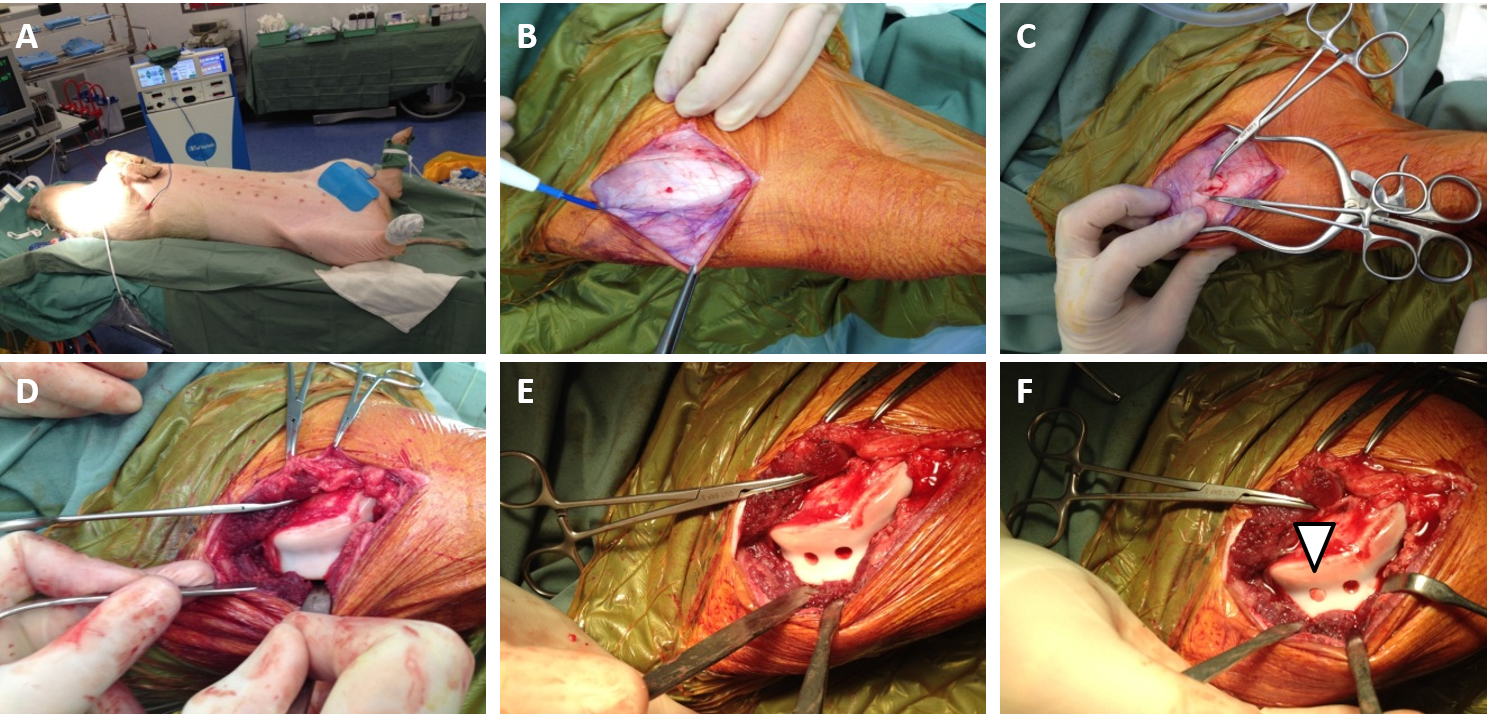


Figure S8. Surgical implantation of the scaffold. (A) The animal is positioned for surgery, (B) parapatellar skin incision, (C) joint capsule opened, (D) patella dislocated laterally to expose the trochlear groove, (E) two 6 mm diameter x 6 mm deep cylindrical defects made in the femoral trochlear groove, (F) acellular scaffold is placed in one site (arrow), the other site treated as empty defect control.

Table S2. Treatment conditions for each subject. Empty = empty defect, MaioRegen™ = commercial scaffold, scaffold = microstructured scaffold, scaffold + cells = microstructured scaffold + allogenic chondrocytes.

| **Subject #** | **Proximal Defect** | **Distal Defect** |
| --- | --- | --- |
| 1 | Empty | Empty |
| 2 | Scaffold + Cells | Scaffold + Cells |
| 3 | MaioRegen™ | MaioRegen™ |
| 4 | Scaffold | Scaffold |
| 5 | Scaffold | Scaffold |
| 6 | MaioRegen™ | Scaffold |
| 7 | Empty | MaioRegen™ |
| 8 | Empty | Scaffold |
| 9 | Scaffold + Cells | Scaffold + Cells |
| 10 | Empty | MaioRegen™ |
| 11 | MaioRegen™ | Empty |
| 12 | Scaffold + Cells | Scaffold + Cells |


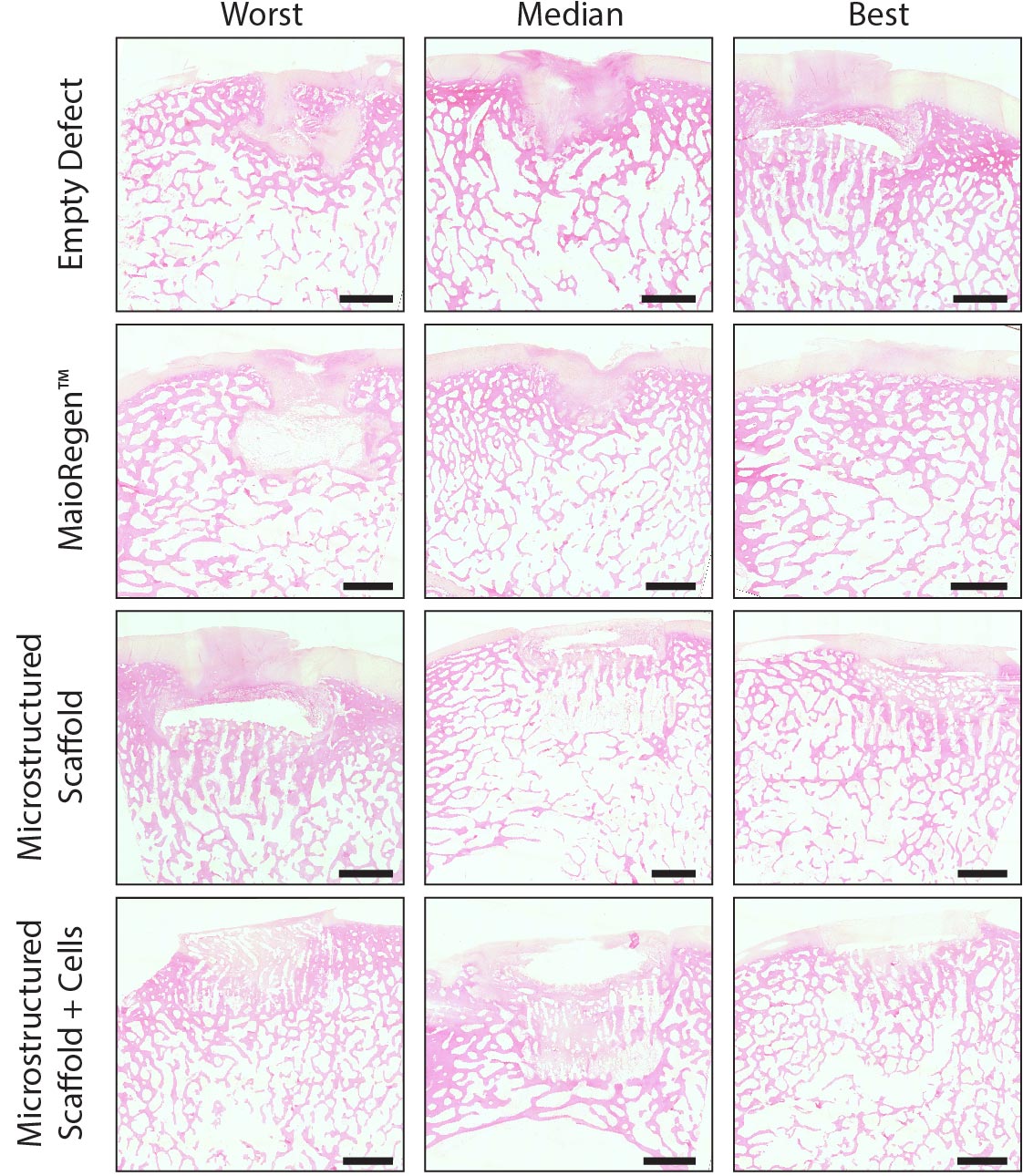


**Figure S9.** Hematoxylin and eosin stained histological sections for each repair type. The qualitative appearance of the stained sections was used to determine the Worst, Median, and Best repair for each condition. Scale bars = 2 mm.


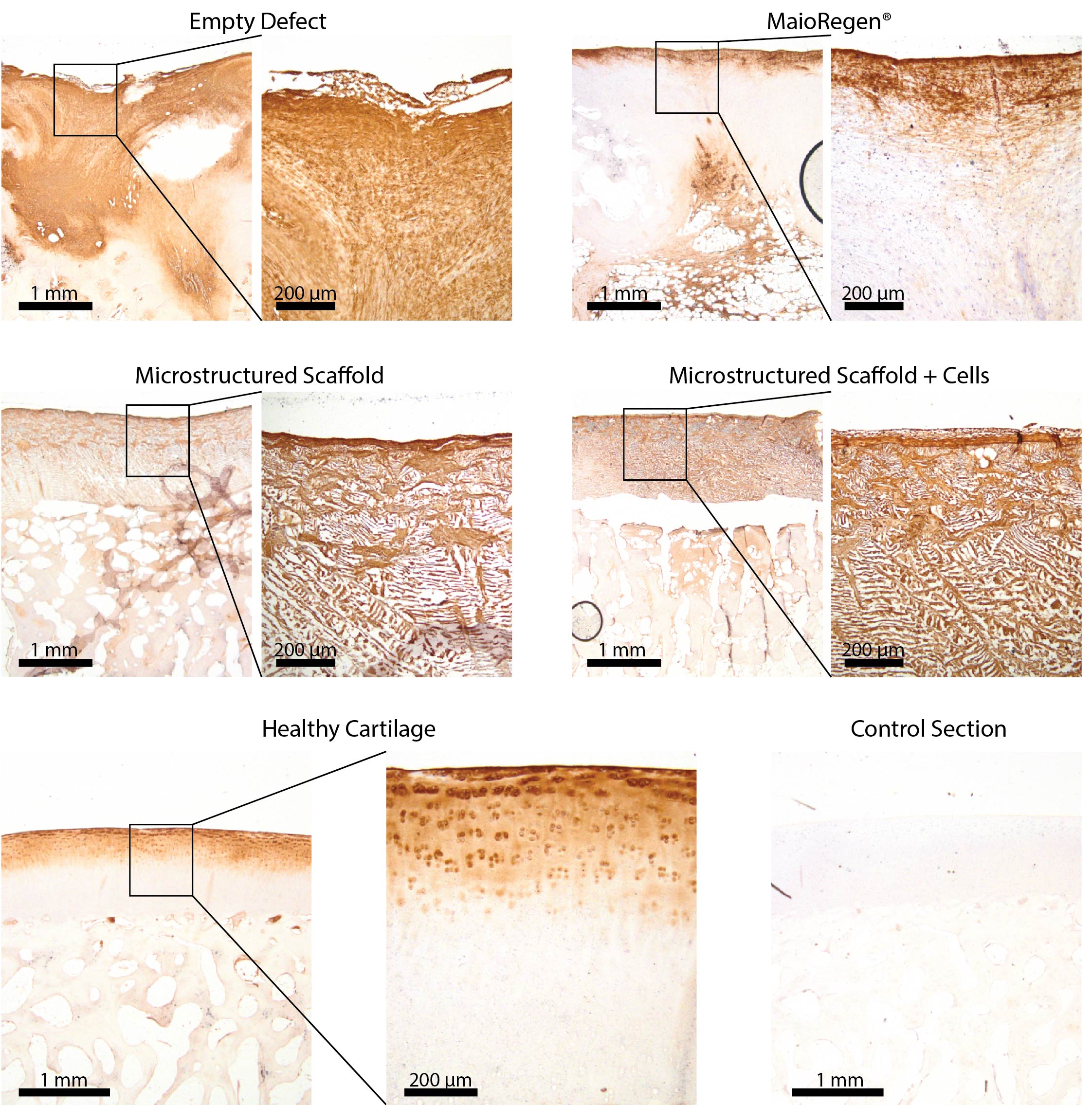


**Figure S10.** Representative IHC sections showing the localization of PRG4 (brown precipitate). The central region of the defect is shown with the top representing the articular surface. A low magnification and high magnification image are shown for each repair type. Normal cartilage is shown in the bottom panels with a negative PRG4 antibody control (bottom right) showing negligible background staining.


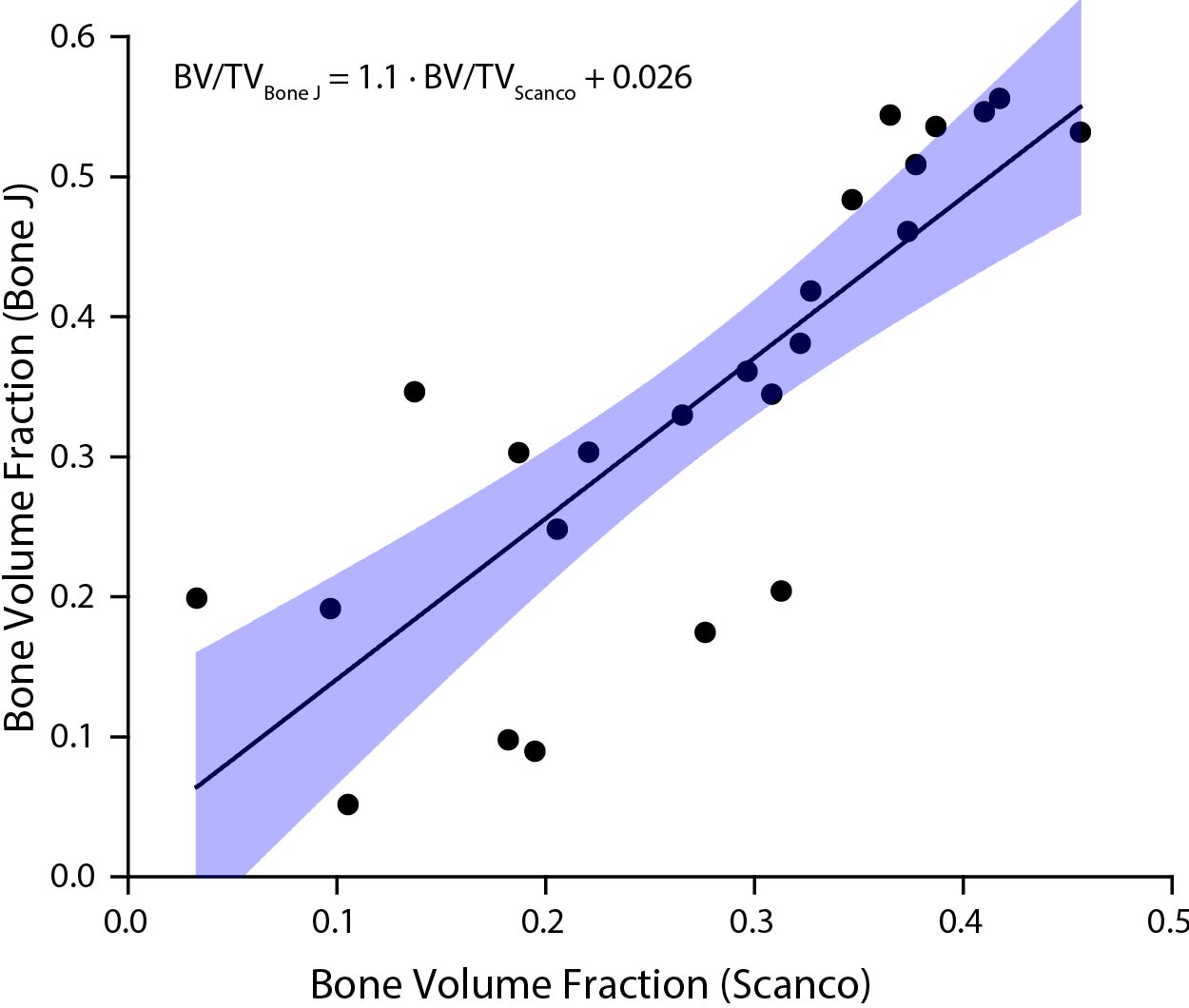


Figure S11. Bone volume fraction calculated using Scanco evaluation software and BoneJ. A linear regression was fit to the data with a 95% confidence interval band shown.


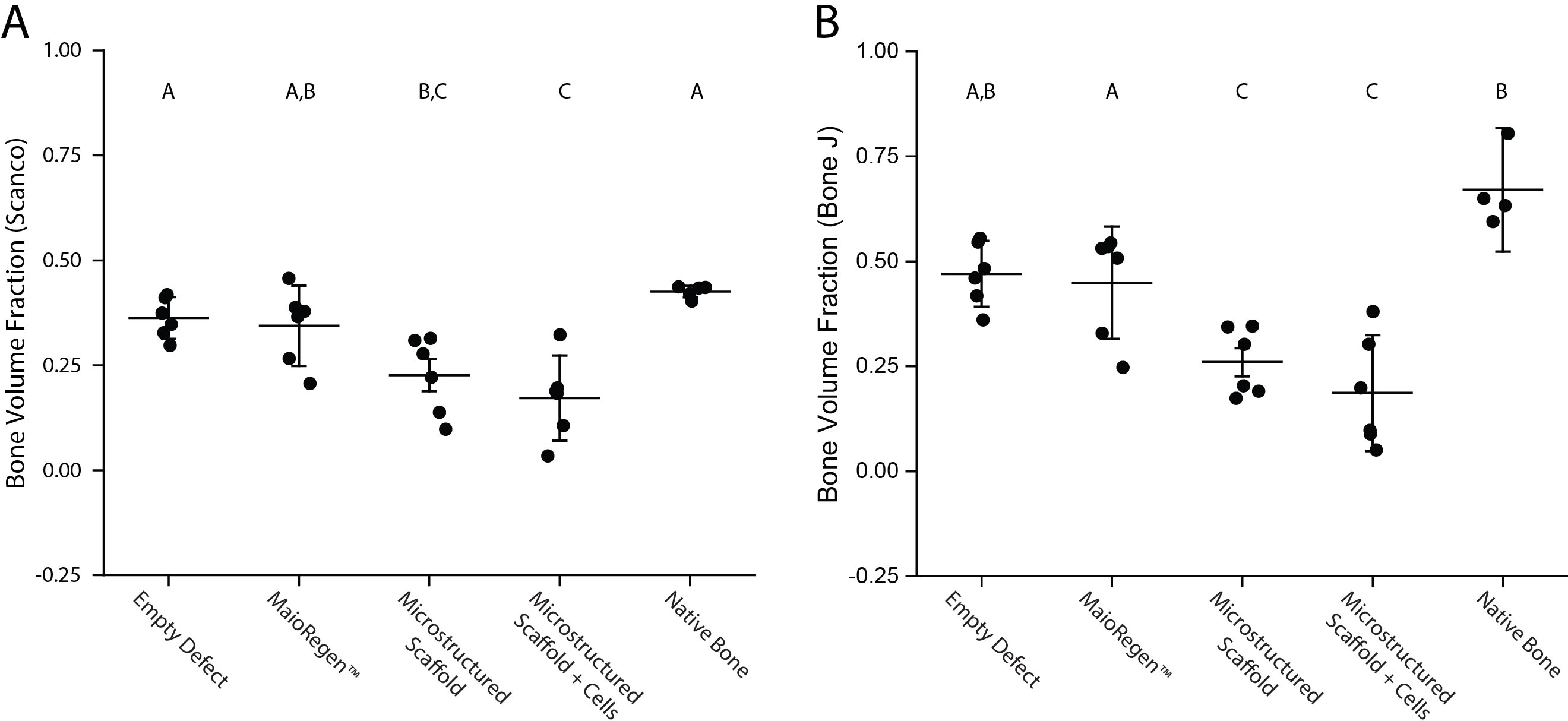


Figure S12. Bone volume fraction was calculated from µCT data using two different methods: (A) Scanco evaluation software and (B) BoneJ. The mean ± 95% confidence interval are shown for each repair type. A one-way ANOVA revealed significant differences across repair types (p < 0.05). Significant differences between samples were detected using Tukey’s method. Repair types that do not share a common letter (A, B, C) are significantly different (e.g., A and B are significantly different). The correlation between the two software packages is shown in Figure S11.


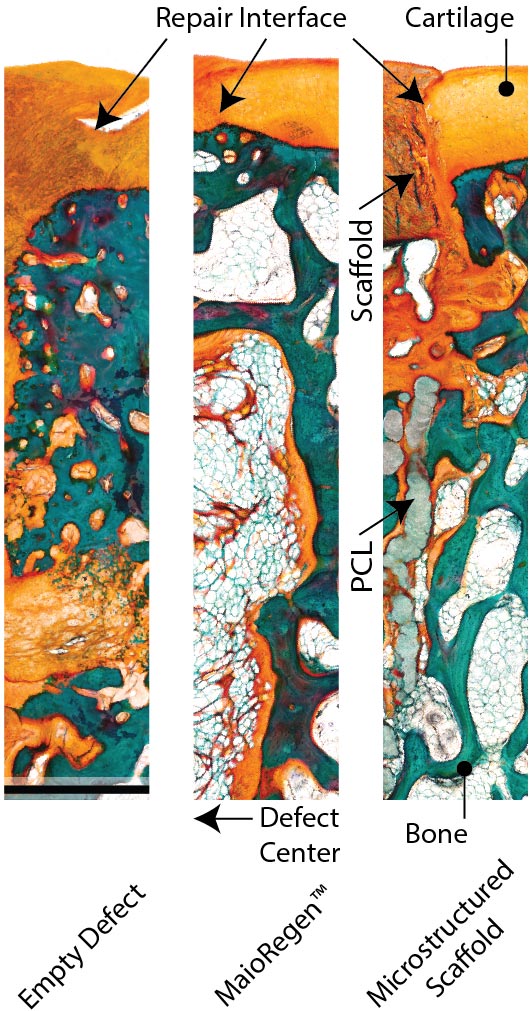


**Figure S13.** Osteochondral repair fronts for empty, MaioRegen™, and microstructured scaffold repair. Reference locations are shown in **Figure 6**. The center of the defect is located to the left, the sliding interface (cartilage-cartilage contact) is positioned at the top, and the subchondral bone is toward the bottom in each image. At 6 months there is clear presence of the scaffold in the chondral region and osteointegration of the PCL lattice in the subchondral bone. Scale bar = 1 mm.


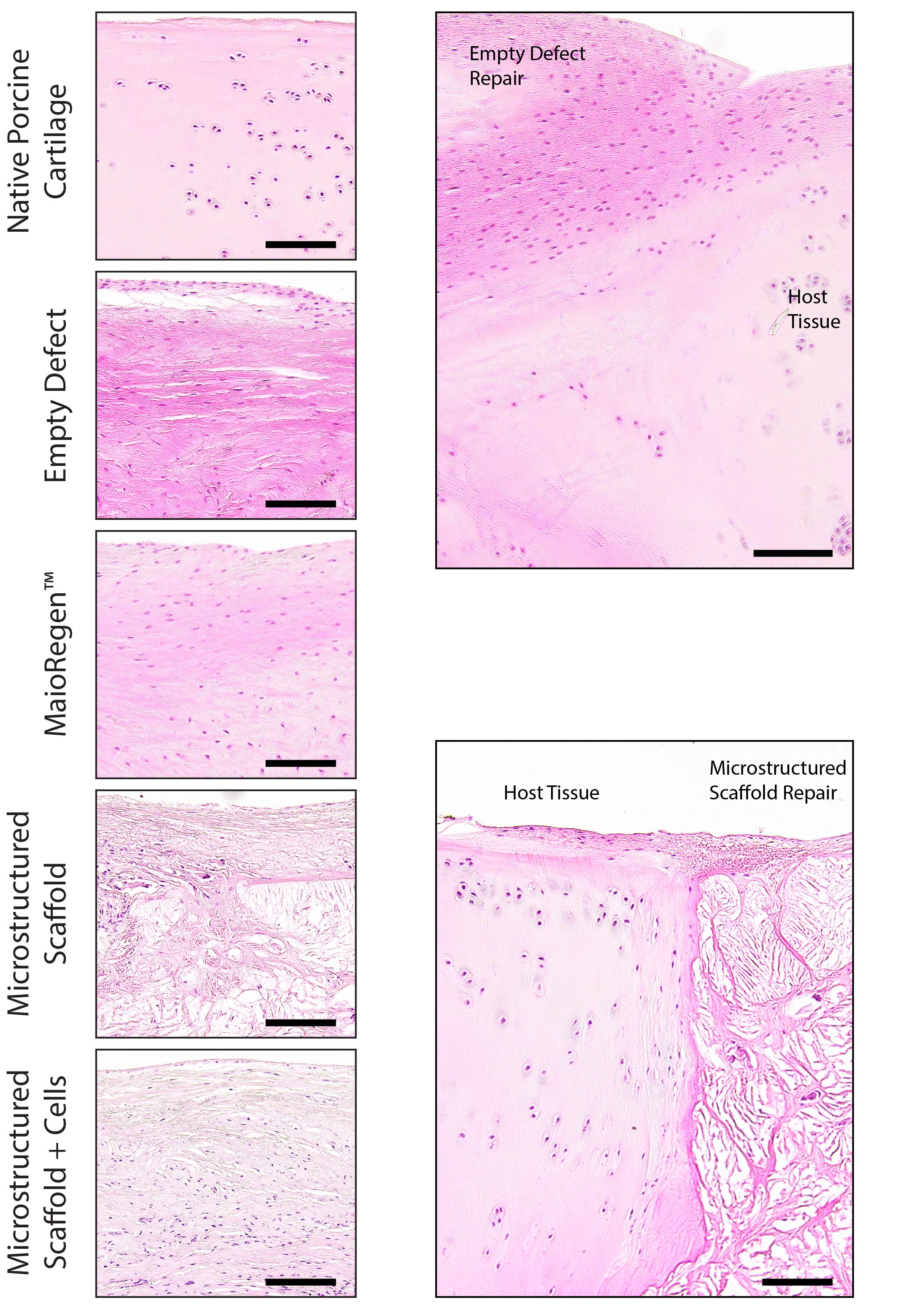


**Figure S14.** Representative hematoxylin and eosin stained histological sections for each repair type. The repair sections are magnified to visualize cell morphology which is part of the histological scoring system. Images of the host-repair interface are shown for the empty and microstructured scaffold repair. Scale bars = 100 μm.


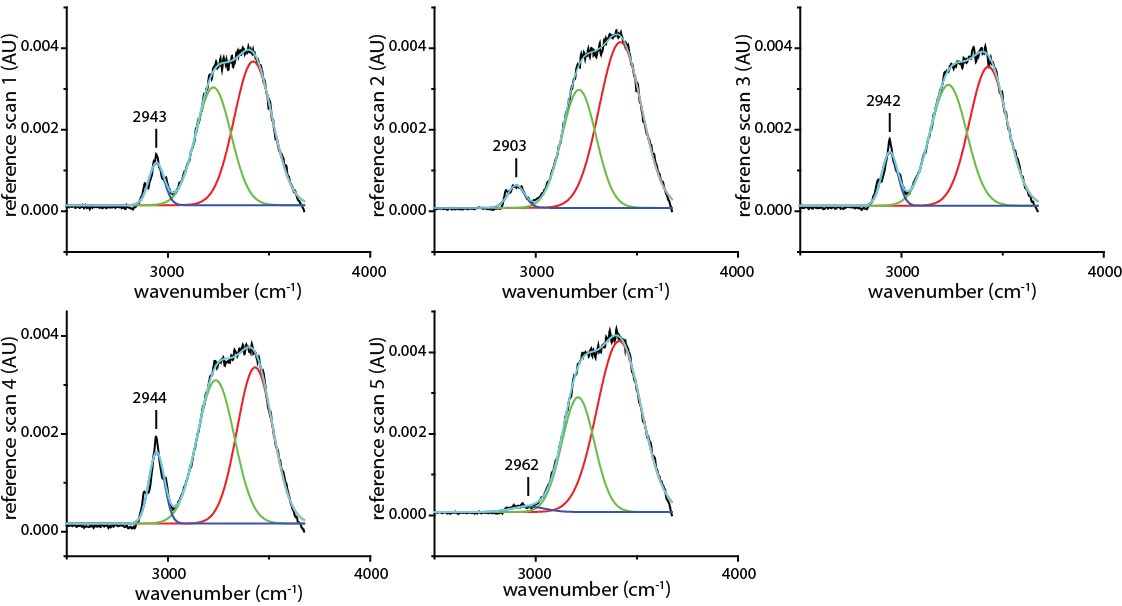


Figure S15. A triple gaussian fit was applied to Raman spectra from 2500 to 3673 cm^-1^ to determine the variation in peak position around 2940 cm^-1^, which is a known Raman shift for lipids and proteins. 5 different scans of healthy porcine cartilage were obtained and fit with this technique. The peak positions are displayed in each plot.


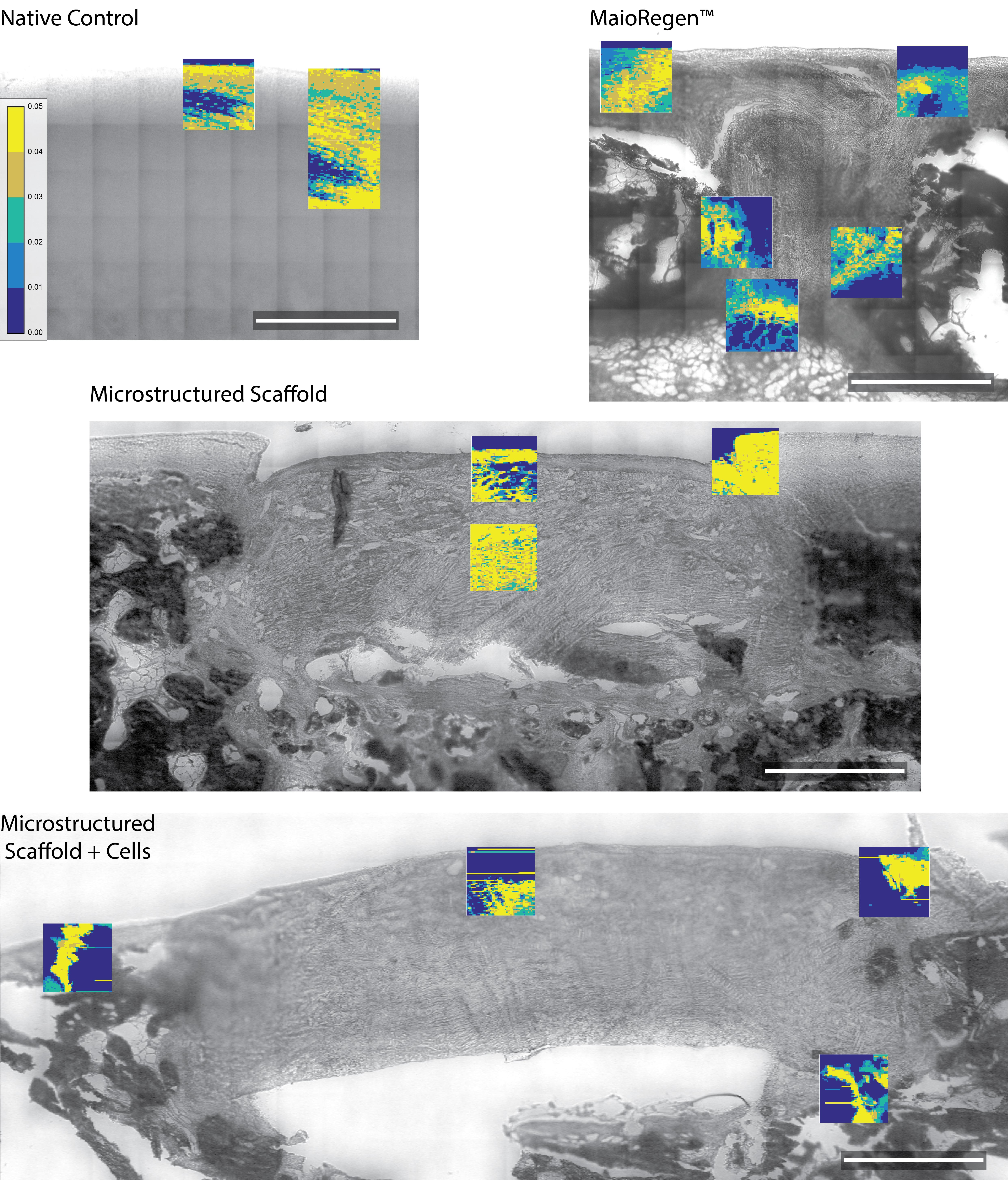


**Figure S16.** Univariate Raman spectra maps are shown for four different repair types. Normalized heat maps show the integrated signal intensity at 2939 ± 22 cm^-1^. Obvious regions of data drop out occurred, possibly due to misalignment between the sample surface and focal plane. Regions of poor signal quality (background and data drop out) were identified using a k-means analysis and excluded from the principal component analysis. Scale bars = 1 mm.


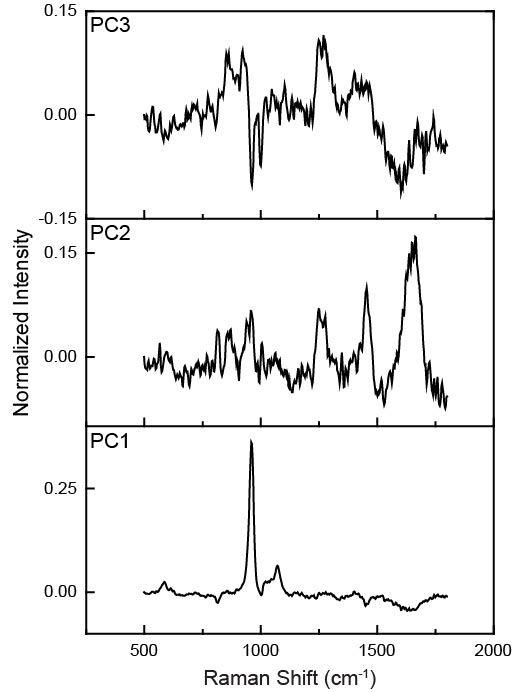


Figure S17. Principal components (PC) 1, 2, and 3.
